# The Future of Tumor Markers: Advancing Early Malignancy Detection Through Omics Technologies, Continuous Monitoring, and Personalized Reference Intervals

**DOI:** 10.3390/biom15071011

**Published:** 2025-07-14

**Authors:** Irem Nur Savas, Abdurrahman Coskun

**Affiliations:** 1 Department of Biochemistry and Molecular Biology, Institute of Health Sciences, Acibadem Mehmet Ali Aydinlar University, Istanbul 34752, Turkey; iremnuryasa@gmail.com; 2 Department of Medical Biochemistry, School of Medicine, Acibadem Mehmet Ali Aydinlar University, Kayisdagi cad., No: 32, Istanbul 34752, Turkey

**Keywords:** genomics, personalized reference interval, proteomics, tumor markers, wearable biosensors

## Abstract

Malignant diseases represent a major global health challenge and are among the leading causes of death worldwide. Accurate early diagnosis is essential for improving outcomes and combating these conditions effectively. Currently, the diagnosis of malignancies relies heavily on radiological imaging and pathological examinations, which are often invasive and not cost-effective. As such, there is a growing need for non-invasive and accessible methods to detect cancer in its early stages. Tumor markers—biomolecules whose levels increase in malignancy and can be measured in blood or other biological tissues and fluids—offer a promising tool. However, the sensitivity and specificity of currently available tumor markers are insufficient for early detection, limiting their use primarily to disease monitoring rather than diagnosis. While ongoing research continues to identify novel tumor markers, the development of more effective early detection strategies requires more than the discovery of new biomarkers. The continuous monitoring of patients and individuals with a high tumor risk and the personalization of tumor marker interpretation are also critical. In this review, we (i) summarize the most commonly used tumor markers, (ii) examine strategies for developing novel biomarkers, particularly through omics technologies, (iii) explore the potential of continuous monitoring using wearable biosensors for early tumor detection, and (iv) discuss approaches to personalizing tumor marker interpretation to support early diagnosis and improve treatment outcomes.

## 1. Introduction

Malignancies have caused profound devastation to human life, emotions, hopes, resources, and economies—few other conditions have had such a widespread and multifaceted impact. According to the World Health Organization, malignant diseases represent the second leading cause of death globally, following cardiovascular diseases [1]. The accurate diagnosis of malignancy constitutes the first and most critical step in combating cancer, because malignant cells tend to grow rapidly and metastasize to distant sites, making the disease significantly more difficult to treat at advanced stages. The doubling times of some common malignant diseases, presented in Table 1, highlight the urgency of early detection and underscore the narrow window available for initiating effective treatment. Therefore, early diagnosis is essential for effective management and improved patient outcomes. Currently, the diagnosis of malignant diseases primarily relies on radiological findings and the examination of patient tissue samples for signs of pathology. However, such approaches may be invasive and potentially harmful, posing significant challenges for broad implementation across the general population. As a result, many common malignancies—such as lung, breast, and gastrointestinal cancers—are often diagnosed at advanced stages, thereby reducing the likelihood of successful radical treatment [2,3,4]. To address this challenge and enable the detection of early malignancies, non-invasive approaches—such as the determination of tumor-specific biomolecules or circulating tumor cells (CTCs)—hold significant potential for early tumor discovery [5,6,7,8,9].

New strategies for the early detection of tumors should be cost-effective, non-invasive, and user-friendly, enabling their integration into routine clinical practice without causing significant discomfort or requiring extensive time. Although many tumor markers are widely employed in clinical practice, most of them lack specificity and their levels can be also elevated in non-malignant conditions (Table 2), often resulting in false positive findings and unnecessary biopsies [24]. Therefore, the identification of novel tumor-specific markers and the development of advanced algorithms to evaluate multiple markers simultaneously are crucial for detecting tumors at the earliest possible stage [25,26]. Given the variable doubling times of tumors (Table 1), effective monitoring is essential. The continuous surveillance of tumor-associated biomarkers may enable us to detect a malignancy at its earliest stage, offering a critical advantage for timely and effective treatment.

Tumor metabolism is also prone to inter-individual variances; therefore, genetic analysis is essential for selecting the most effective drugs or treatment strategies [43,44,45,46,47]. Both the interpretation of tumor marker levels and the assessment of treatment efficacy should be based on the individual’s clinical data rather than population means. In other words, a personalized approach is required—one that not only tailors the interpretation of tumor markers and treatment plans but also evaluates therapeutic effectiveness using the individual’s unique biological profile.

In this review, we (i) briefly overview the most commonly used tumor markers, (ii) present strategies for the development of novel markers, (iii) explore the potential of continuous monitoring to detect tumors at an early stage, and (iv) discuss how the interpretation of tumor markers can be personalized to facilitate early detection and thereby enable more effective treatment.

## 2. Tumor Markers

Tumor markers are biomolecules that are overproduced or structurally altered as a cause or consequence of malignant processes. Tumor markers can be found intracellularly or extracellularly, the latter are often released into the circulation. Many of them can also be detected in other body fluids such as interstitial fluid (ISF), urine, seminal fluid, tears, and saliva. Tumor-associated molecules exhibit wide structural variation—they are proteins (peptides, enzymes, transporters, and hormones), carbohydrates, or even lipids. Additionally, CTCs have gained attention for their diagnostic and prognostic potential in cancer. This review focuses primarily on tumor markers that can be detected in blood or other body fluids.

### 2.1. Proteins

**Figure 1:**
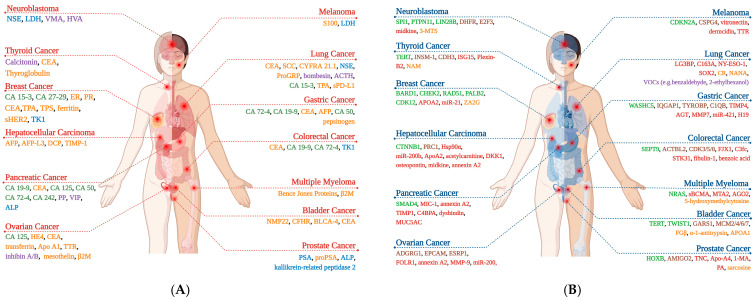
Biomarkers used as established tumor markers and potential tumor markers under investigation. (**A**) Frequently used tumor markers. Color coding: orange (protein, Section 2.1), blue (enzyme, Section 2.2), purple (hormone, Section 2.3), and green (carbohydrate antigen, Section 2.4). Created in BioRender. Coskun, A. (2025) https://BioRender.com/ow3kws3. (**B**) Novel tumor markers. Color coding: green (genetic alterations), brown (tissue), red (blood), orange (urine), and purple (breath). Created in BioRender. Coskun, A. (2025) https://BioRender.com/2cof13n. Except for ovarian and prostate cancer, the cancers shown are not gender-specific. The associated references for novel tumor markers are provided in the Supplementary Materials, Tables S1–S4.

The majority of tumor markers used in clinical practice are proteins. Although enzymes are proteins and several hormones are peptides or proteins, they are often classified separately due to their distinct biological functions. Proteins in use as tumor markers include oncofetal proteins such as alpha-fetoprotein (AFP) and carcinoembryonic antigen (CEA); protein fragments such as tissue polypeptide antigen and cytokeratin-19 fragment antigen 21-1 (CYFRA 21-1); and various immunoglobulins including Bence Jones proteins, as well as other proteins such as β2-microglobulin, chromogranin A, human epididymis protein 4 (HE4), thyroglobulin, squamous cell carcinoma antigen (SCC), and S100 protein (Figure 1A) [24].

Although the abovementioned markers are primarily used for the monitoring of cancer patients, a few have relatively higher diagnostic value, such as AFP and Bence Jones proteins [24]. Several proteins including ferritin, mesothelin, and bladder cancer–specific nuclear matrix protein 4 are considered potential tumor markers. However, their clinical utility as tumor markers is limited as they are still under investigation [48,49,50]. Additionally, certain cancer-associated membrane proteins may be released into the circulation through a proteolytic cleavage, and their soluble forms are also used as tumor markers, including sHER2 and sPD-L1 [51,52,53] (Figure 1A).

AFP and CEA are the most commonly used protein tumor markers and are briefly described in the following text.

#### 2.1.1. Alpha-Fetoprotein (AFP)

AFP is an oncofetal protein and is the most widely used biomarker for hepatocellular carcinoma (HCC) [54]. AFP, an analog of albumin, is the predominant serum protein during the first trimester of gestation and declines to trace levels by 18 months after birth [55,56]. AFP serves as a carrier for various molecules, including retinoids and steroids, similarly to serum albumin. The serum AFP level in healthy adults is typically below 10 µg/L and elevated levels are primarily associated with HCC or non-seminomatous germ cell tumors [55]. Not many tumor markers are recommended for cancer screening and AFP is among them, alongside CA 125, human chorionic gonadotropin (hCG), and a few others [24]. AFP is used in combination with ultrasound for screening high-risk individuals with chronic hepatitis, including those infected with hepatitis B (HBV) or hepatitis C virus (HCV) or those with advanced liver fibrosis [24]. For early stage HCC, an AFP and ultrasound combination exhibits a detection rate of 63%, prompting the search for additional markers [57,58]. It seems that levels of AFP-L3 (an isoform of AFP) and des-gamma-carboxyprothrombin (DCP), also known as prothrombin induced by vitamin K absence-II (PIVKA-II), are not correlated with AFP levels. Hence, AFP-L3 and DCP may be elevated in HCC patients even when AFP remains within the normal range, supporting their role as complementary diagnostic markers [58,59]. In the most recent study [60], the diagnostic performance of a biomarker panel including AFP, AFP-L3, DCP, and CA 19-9 was evaluated in liver diseases. Receiver operating characteristic (ROC) curve analysis showed how the combination of the four markers outperformed each individual marker in distinguishing liver cancer from benign liver diseases (area under the ROC curve, AUC = 0.85) or from healthy controls (AUC = 0.95) [60]. More large-scale studies are needed to establish the role of AFP-L3 and DCP in clinical practice.

#### 2.1.2. Carcinoembryonic Antigen (CEA)

CEA was initially identified in human colorectal cancer (CRC) [30]. CEA is an embryonal glycoprotein found in fetal tissues, primarily in the gastrointestinal tract [61]. The major role of CEA is in cell adhesion. Its production ceases before birth, resulting in only minimal levels in the blood of healthy adults (~3 ng/mL in non-smokers, ~5 ng/mL in smokers) [55]. Only 30–50% of CRC patients present with elevated CEA levels, so its use alone does not provide a reliable diagnosis [24]. However, after confirmed cancer diagnosis, CEA is a valuable biomarker in monitoring treatment response, detecting tumor recurrence, and guiding follow-up strategies [62,63]. More frequent CEA measurements in post-operative CRC patients contribute to overall survival, and it is recommended to repeat measurements every 3–6 months for 5 years [24]. Although primarily associated with CRC, elevated CEA levels can also be useful in the follow-up of gastric, lung, and breast cancer patients [24]. A considerable number of studies have reported that the inclusion of CEA in diagnostic panels comprising various markers increases their diagnostic accuracy [64,65,66,67,68,69,70]. As a supporting example, a panel consisting of CEA, CYFRA 21-1, neuron-specific enolase (NSE), SCC, and ProGRP demonstrated higher diagnostic sensitivity (88,5%) and specificity (82%) than each marker alone in detecting lung cancer in patients with symptoms suggestive of this cancer [70]. Therefore, despite its limited specificity, CEA remains an essential biomarker with significant clinical relevance.

### 2.2. Enzymes

In oncology, enzymes are generally used as non-specific tumor markers, since they reflect tissue damage rather than indicating a specific malignancy. As an exception to this, tissue-specific isoenzymes are more typical for certain tumors. For example, prostate-specific antigen (PSA) is specific to prostate tissue and is consequently used in the management of prostate cancer and NSE is highly specific for tumors of neuroendocrine origin (also tumors with neuroendocrine differentiation) [71,72]. Other enzyme tumor markers include lactate dehydrogenase, alkaline phosphatase, thymidine Kinase-1, and kallikrein-related peptidase 2 (Figure 1A) [73,74,75].

PSA stands out among all enzyme tumor markers due to its unique specificity for the prostate, and it is described below.

#### Prostate-Specific Antigen (PSA)

PSA, first isolated from prostate tissue [36], is a proteolytic enzyme belonging to the kallikrein serine protease family. Physiologically, PSA facilitates the liquefaction of seminal fluid by proteolytically cleaving gel-forming proteins, thereby enhancing sperm motility. When the prostatic epithelial tissue is disrupted, increased amounts of PSA are released into the circulation. This elevation may result from a prostate cancer, but can also occur due to benign prostatic diseases [76]. PSA is widely used for monitoring prostate cancer. Although a cut-off value of 4 ng/mL is useful in diagnostic evaluation, it does not provide sufficient sensitivity [76]. Various strategies have been developed to improve the diagnostic accuracy of PSA and to reduce the number of unnecessary biopsies (~60% of all cases) [77]. Initial strategies included PSA density (total PSA/prostate volume) measurements, and PSA density over 0.2 ng/mL/cm^3^ has been associated with a significantly increased risk of malignancy [78]. On the other hand, statistical prediction models that incorporate various molecular forms of PSA (such as free PSA, proPSA, and intact PSA) and kallikrein-related peptidase 2, along with patient age and digital rectal examination, have demonstrated enhanced diagnostic accuracy (up to an AUC of 0.8) compared to total PSA alone (AUC ~0.6). More recently, Kachuri et al. proposed adjusting PSA levels based on individual genetic variation [79]. In a large multi-ancestry study, they developed a PSA polygenic score that accounted for approximately 10% of PSA variability. Genetically adjusted PSA levels reduced unnecessary prostate biopsies by up to 31%, although further studies are needed to refine their clinical utility.

### 2.3. Hormones

Hormones, resulting from either excessive production by the original endocrine tissue or ectopic synthesis by non-endocrine tissues, can also serve as cancer biomarkers [55]. Calcitonin is the most frequently utilized hormone tumor marker (see below). Another hormone, hCG, primarily associated with germ cell tumors, has a specific clinical application in the screening of gestational trophoblastic neoplasia, a rare malignancy that may develop following a molar pregnancy [80]. Other hormone-based tumor markers include bombesin, prolactin, and adrenocorticotropic hormone; catecholamines, including homovanillic acid and vanillymandelic acid; and also gut hormones, such as vasoactive intestinal peptide and pancreatic polypeptide (Figure 1A) [24,55].

#### Calcitonin

The first association between the hormone calcitonin and cancer was established when elevated levels were detected in patients with medullary thyroid carcinoma (MTC) [33,81]. It is a polypeptide hormone primarily involved in calcium homeostasis and mainly produced by the thyroid gland as part of a larger precursor molecule procalcitonin (ProCT). In MTC patients, calcitonin has a well-established role in assessing prognosis and is also routinely used with CEA for pre- and post-operative follow-up. Calcitonin levels exceeding 50–100 ng/L have diagnostic potential for MTC [82]; however, calcitonin as a cancer biomarker has a limited value due to certain drawbacks. Although calcitonin levels in blood may discriminate between cancer and healthy populations, the primary issues are the absence of established cut-off values and the short half-life of calcitonin in serum (15–40 min). Moreover, calcitonin’s daily fluctuations pose an additional analytical constraint to its wider use as a cancer biomarker [81,82]. Studies focusing on determining age- and gender-specific cut-off values are expected to increase the diagnostic usefulness of calcitonin for MTC [83]. Fortunately, compared to calcitonin, ProCT exhibits a longer half-life (20–24 h), thus providing a greater analytical stability [82]. A meta-analysis reported that ProCT measurement provided high pooled sensitivity (90%) and specificity (100%) in detecting MTC, making it a promising alternative diagnostic marker [84]. However, no comparison between calcitonin and ProCT was included, and further studies are warranted to confirm the clinical utility of the latter [84].

### 2.4. Carbohydrate Antigens

Tumor-associated carbohydrate antigens are glycans covalently bound to proteins (glycoproteins) or lipids (glycolipids), which are expressed on the surface of the tumor cells [26,55]. While these structures are absent or present at very low levels on normal cells, they are often overexpressed or structurally altered in malignant cells, leading to aberrant glycan profiles. Tumor-associated carbohydrate antigens such as CA 19-9, CA 125, CA 15-3, CA 72-4, and CA 27-29 are predominantly found among mucins, which are heavily glycosylated carrier glycoproteins [26,55]. These antigens are shed into the circulation during malignant processes and their levels are used clinically in monitoring malignancies. Due to their generally limited specificity, the diagnostic use of these biomarkers often requires additional methods and is typically interpreted alongside imaging techniques. Among the commonly used carbohydrate antigens, CA 19-9, CA 15-3, and CA 125 are discussed below.

#### 2.4.1. Carbohydrate Antigen 19-9

CA 19-9, also known as sialyl Lewis antigen A, is the best tumor marker available for monitoring pancreatic cancer [85]. The presence of the Lewis antigen is required for the biosynthesis of CA 19-9, therefore Lewis antigen-negative individuals (5–10% of the population) can produce minimal amounts of CA 19-9, which must be taken into consideration when evaluating the usefulness of this cancer biomarker. CA 19-9 has diagnostic utility in pancreatic cancer with a sensitivity of 79% and a specificity of 82% [86]. In the early stages of pancreatic cancer, the sensitivity has been reported as 76.1% [85]. Many strategies have been developed to increase the diagnostic potential of CA 19-9 [86,87]. One of the recommended approaches is the combination of CA 19-9 with other commonly used tumor markers, including CEA, CA 125, and CA 242, as well as novel markers such as macrophage inhibitory cytokine-1, mucin 5AC (MUC5AC) [86,87]. For instance, combining CA 19-9 with MUC5AC significantly enhances predictive diagnostic performance (AUC = 0.91) compared to CA 19-9 alone (AUC = 0.61) [88]. Another strategy involves assessing the genetic status of patients, specifically genes coding for proteins associated with CA 19-9 biosynthesis. Using genotype-dependent cut-off values based on Lewis-negative and secretor-negative status improved the sensitivity of CA 19-9 for detecting early stage pancreatic cancer from 76.1% to 87.2% [85]. Large-scale studies will be useful for supporting the clinical and diagnostic applications of CA 19-9.

#### 2.4.2. Cancer Antigen 125

CA 125 is a well-established tumor marker that represents an epitope derived from the transmembrane mucin 16 (MUC 16) [89]. Elevated CA 125 levels (>35 U/mL) are most commonly associated with epithelial ovarian cancer, but they may also be observed in other malignancies as well as in various non-malignant conditions (Table 2). Although it is not recommended for routine screening in healthy individuals, CA 125 has potential in screening high-risk women, particularly those with a family history of ovarian and breast cancer, or women identified as *BRCA1/2* mutation carriers [90]. Currently, CA 125 is the most effective serum marker for ovarian cancer, though HE4 demonstrates a comparable diagnostic sensitivity (75%) and specificity (90%) [90,91]. Nevertheless, considerable efforts are being made to identify novel biomarkers with an improved diagnostic performance [89]. A comprehensive study evaluated the diagnostic value of 92 potential biomarkers [91]. Among these, eight candidates with relatively high diagnostic potential were further assessed in combination with CA 125, and a CA 125 and ADAM8 protein combination yielded the best diagnostic performance. However, this combination resulted in only a modest improvement (2%) in diagnostic accuracy compared to CA 125 alone [91]. More recently, it has been reported that assessing the mutational status and glycosylation extent of MUC 16, alongside CA 125 measurements, may offer a more reliable tool for the early diagnosis of ovarian cancer [89].

#### 2.4.3. Cancer Antigen 15-3

CA 15-3 is a glycoprotein fragment derived from MUC1, the latter being a protein product of the breast cancer-associated *MUC1* gene. CA 15-3 is the most widely utilized serum biomarker for breast cancer [26,92]. Its primary clinical application is in the assessment of metastatic disease, where it is interpreted in conjunction with imaging and clinical findings [93]. Elevated levels of CA 15-3 (>40–50 U/mL) at cancer diagnosis have been associated with poor prognosis and poor survival outcomes, probably reflecting its ability to detect clinically silent micro-metastases [24,93,94,95]. However, in early stages, CA 15-3 levels generally remain within normal limits (≤30 U/mL), thereby limiting its effectiveness for early detection [24,93]. To sum up, as in many other types of cancer, a reliable tumor marker for early breast cancer detection is still lacking.

Recently, Sekacheva et al. reported that the combined use of the novel biomarker CA-62 with CA 15-3 significantly enhances diagnostic accuracy in breast cancer [96]. Unlike CA 15-3, CA-62 has been identified as an epithelial carcinoma marker associated with abnormal cell proliferation and has been elevated in early stage breast cancer. The diagnostic accuracy of CA-62 (97%) and CA 15-3 (40%) in stage I disease has been reported [96]. Another study evaluating the combined use of these two markers demonstrated 75% sensitivity and 100% specificity for stage I breast cancer, underscoring their complementary diagnostic potential [97]. Notably, while the diagnostic performance of CA-62 declines in advanced stages, that of CA 15-3 improves, suggesting that their simultaneous use may offer diagnostic benefit in breast cancer detection [96,97]. Nevertheless, further studies are required to validate the clinical applicability of the CA-62 + CA 15-3 diagnostic combination.

### 2.5. Circulating Tumor Cells (CTCs)

CTCs detach from the primary tumor or metastases and enter the circulation, thus contributing to metastasis [98]. Ever since their potential for early cancer diagnosis, as well as for prognosis assessment and metastasis monitoring, has been demonstrated, CTCs have been extensively investigated for their clinical applications in oncology [99,100,101,102,103]. In an early stage cancer, CTCs are rare in blood (<5 CTCs per 7.5 mL of blood), with a short half-life ranging from 1 to 2.4 h [104]. However, their concentration may serve as an informative tumor marker [98]. As an example, in non-small cell lung cancer (NSCLC), five CTCs in 7.5 mL of blood has been used as a baseline value, and more CTCs have been associated with a worse prognosis [105]. Another study reported that abnormal lung imaging results and a CTC value over 25 per 7.5 mL can distinguish lung cancer from benign lesions and has the potential to be used for screening purposes [106]. In breast cancer, five CTCs per 7.5 mL has been suggested as a cut-off for a metastatic stage, while one CTC/7.5 mL has usually been detected in the localized disease [107]. According to several large prospective studies and meta-analyses, patients with high CTC counts in the blood generally have an extremely poor prognosis [100,106]. In addition to the quantitative assessment of CTCs, an emerging application is their use in liquid biopsy, an innovative, non-invasive alternative to tissue biopsies. Liquid biopsies are a source of tumor-derived nucleotides or proteins, and can be used as a source for advanced omics analyses (see Section 3.1.3).

## 3. Strategies for Early Detection of Malignancies

Despite the availability of numerous tumor markers (Figure 1A), current clinical practice does not enable the reliable early detection of tumors using these markers. Therefore, new strategies are essential to identify and clinically validate novel tumor markers for routine application. These strategies should be built upon three key cornerstones: (i) the discovery and development of novel tumor markers through omics technologies, (ii) the continuous monitoring of individuals using wearable biosensors, and (iii) the personalization of individual data to facilitate tumor detection in an early stage, as detailed below.

### 3.1. Omics Technologies in Tumor Marker Discovery: Opportunities and Challenges in Malignancy Diagnosis

To detect malignancies at an early stage, the metabolic activities of tumor cells—which serve as traces indicating the presence of tumors—should first be identified. The basic structural units of living systems are biomolecules; therefore, the transformation of a normal cell into a malignant one is accompanied by changes in the activities of biomolecules or alterations in their abundance. During the malignant transformation of a cell, some silent genes may become activated, leading to an increased expression of specific proteins and/or elevated biosynthesis or degradation of other biomolecules such as oligosaccharides, lipids, and metabolites. This way, certain biomolecules may appear at much greater concentrations than in healthy individuals. It is not enough to discover novel tumor-specific biomolecules, they must be clinically validated. Unfortunately, despite the discovery of dozens of cancer-associated biomolecules, a small number of them have been clinically validated as tumor markers. Moreover, most of these were investigated before the advent of the omics era (Table 2).

The first tumor marker to be identified was the Bence Jones protein, discovered in 1847, which is still widely used today in the diagnosis of multiple myeloma [27,108]. In 1927, hCG was associated with pregnancy and only later was it recognized as a tumor marker for germ cell tumors and gestational trophoblastic disease [28]. These two proteins represent the **first generation of tumor markers** that appeared in the period **from 1847 to 1960 [109]**. **Second-generation tumor markers (1960–1970)** [109] emerged following a pivotal discovery of AFP in mice inoculated with liver cancer cells (in 1963) [29]. During these years, advancements in analytical technologies, most notably the development of the radioimmunoassay, enabled large-scale studies. In 1975, elevated serum CEA levels were detected in cancer patients [30]. Shortly thereafter, immunometric assays based on monoclonal antibody technologies greatly accelerated the discovery of tumor markers [24]. This progress led to the discovery of many carbohydrate antigens such as CA 125, CA 15-3, and CA 19-9, also known as the **third generation of tumor markers (1970–1980), [109]** which became essential tools in cancer management. The **fourth generation of tumor markers (1980–1990)** comprised genetic markers including *BRCA1/2*, *HER2*, and *TP53*, all of which had already been introduced into clinical practice [26]. Between 1990 and 2004, coinciding with the Human Genome Project, a significant number of oncogenes, proto-oncogenes, and tumor suppressor genes were identified [26]. Thus, for many years, tumor marker discovery efforts focused primarily on individual molecules, single genes, and specific molecular pathways. After 2005, the expansion of scientific knowledge and the acceleration of technological developments (such as high-throughput sequencing, micro-array technologies, quantitative mass spectrometry, and computational bioinformatics platforms) facilitated the emergence of omics technologies and shifted tumor marker research into a more comprehensive and innovative phase [26].

Ironically, despite great expectations, omics technologies have so far not yielded the anticipated tumor-specific and sensitive biomarkers suitable for early stage detection in clinical laboratories. However, this does not preclude the possibility that such biomarkers or panels thereof will eventually be identified, and there is hope that omics technologies will ultimately fulfill this promise. **Fifth-generation tumor markers (2005–present)** predominantly comprise omics-based biomarkers, which will be discussed in this section.

#### 3.1.1. Application of Omics Approaches for Novel Tumor Marker Identification

The term “-omics” refers to the comprehensive analysis of various molecular components within biological systems [110]. From an oncological perspective, major omics technologies are used to gain a comprehensive understanding of tumor biology. Among them, genomics serves as a large-scale analysis of genes to identify genetic alterations driving tumor development. Transcriptomics is employed for the identification of tumor-specific gene expression profiles and the molecular classification of tumors. Proteomics is used to elucidate changes in the expression of thousands of proteins and, when possible, changes in protein functions resulting from tumor progression. Finally, metabolomics and lipidomics are employed to assess dynamic metabolic processes in cancer and to characterize distinct metabolic profiles influenced by a wide range of factors, including genetic alterations and environmental conditions [25,111,112]. Each of these approaches holds strong potential for improving early cancer diagnosis, whether by enabling the identification of tumor-specific genetic and molecular profiles or, more specifically, by facilitating the discovery of novel tumor markers.

In the omics-based approaches, the process of translating a newly discovered biomarker from initial identification to clinical application involves four main stages: discovery, analytical validation, an assessment of clinical utility, and clinical implementation, along with several intermediate steps [113]. These include precise disease characterization, sample collection, high-throughput data acquisition, data analysis, the identification of candidate biomarkers, independent validation, and determining the validity of the biomarker across large populations [113]. To date, many candidate tumor markers have been discovered and presented by utilizing advanced omics technologies (Figure 1B). Next-generation sequencing, which has accelerated the advancement of genomic technologies, has enabled the simultaneous detection of multiple genetic alterations and the identification of genetic profiles associated with specific tumors [114]. For instance, beyond the well-known *BRCA* mutations in breast cancer, alterations in genes such as *BARD1*, and pathogenic variants of *RAD51C* and *RAD51D*, reported to be associated with increased breast cancer risk, have also been identified [114]. On the other hand, *TERT* promoter mutations, which contribute to early tumorigenesis by promoting telomerase activation, have been associated with more than 50 malignancies through comprehensive genomic analyses [115,116,117,118]. Moreover, *TERT*-associated alterations manifest as elevated mRNA expression levels, and their potential as tumor biomarkers for early diagnosis and prognosis has been demonstrated across various cancers, including bladder cancer and glioma [115,116,117,118].

The availability of The Cancer Genome Atlas, Human Protein Atlas, and other large-scale databases enables in silico approaches for screening gene expression and protein profiles, identifying tumor-specific genetic and molecular signatures, and detecting novel biomarker candidates [119,120]. These platforms offer an alternative to conventional sample collection and experimental analysis. A large number of studies are currently being conducted using these data-driven strategies, with ongoing efforts to identify potential tumor biomarkers for possible use in diagnostic settings. As an example, Zalfa et al. identified a panel of 41 genes that were found to be expressed up to 300-fold higher in ovarian cancer compared to non-malignant samples, based on a database-driven transcriptomic analysis. Among them, a model consisting of eight genes (including *ADGRG1*, *EPCAM*, and *ESRP1*) could distinguish ovarian serous cystadenocarcinoma (n = 42) from healthy ovarian tissues (n = 3) with sensitivity and specificity reaching 100%. The RNA expression levels of these genes were confirmed in an independent sample set. Expanding the sample size may contribute to the further evaluation of their diagnostic potential [121].

Despite the vast amount of genetic and transcriptomic data that has already been collected, this information cannot be directly translated into protein-level expression. Proteomic studies, leveraging new technologies, have uncovered the early diagnostic potential of various proteins. For instance, many proteins, including osteopontin, midkine, galectin-3, and annexin A2, were initially discovered before the emergence of omics technologies, yet large-scale proteomic analyses later revealed their potential for the early detection of malignancy [122,123,124]. Elevated levels of osteopontin have been associated with poor prognosis across multiple malignancies, including breast, pancreatic, and bladder cancers. There were several reports of osteopontin exhibiting diagnostic sensitivity comparable or even superior to AFP in HCC [125,126]. However, similar to the traditional tumor markers, osteopontin can be elevated in certain benign conditions, thus posing a significant limitation [127,128]. Additionally, proteomic studies have facilitated the discovery of novel tumor markers, by identifying and/or quantifying differentially expressed proteins. For instance, cyclin-dependent kinase 3 and actin beta-like 2 have been presented as potential tumor markers detected in tissue samples [129], while apolipoprotein A2 [130], heatshock protein 90α [131], dickkopf-1 [132], dysbindin [133], MUC5AC [134], TIMP-1 [135], vitronectin, dermcidin [136], transthyretin, and angiotensinogen [137] have been identified in serum. Malignancies associated with the listed proteins are displayed in Figure 1B.

Although examples of such protein biomarkers can be substantially expanded across different cancer types, the overall diagnostic potential of these emerging markers rarely exceeds that of conventional biomarkers currently used in clinical practice. The main reason is that the superiority of novel protein biomarkers is typically demonstrated in small-scale studies with limited patient cohorts. In response to these limitations, a growing body of research has focused on the combined use of established biomarkers and newly identified candidates derived from omics-based analyses, aiming to improve diagnostic performance through complementary biomarker panels. As an example, Gao et al. identified, through proteomics, a novel bile biomarker, clusterin (CLU), to improve the diagnosis of cholangiocarcinoma. The reported sensitivity of CLU was 73.6% and its specificity was 90.1%. Subsequently, a seven-marker diagnostic panel was developed by combining CLU with serum CA 19-9 and five additional biochemical parameters, including indirect bilirubin and gamma-glutamyl transferase. This panel demonstrated improved diagnostic performance, achieving 90.3% sensitivity in an independent validation cohort [138]. To facilitate the clinical applicability of the panel, the researchers created a user-friendly online model that displays the diagnostic prediction with a percentage probability [138].

On the other hand, individual metabolomes reflect the individual conditions in a highly personalized and dynamic way, as they result from a wide variety of factors, such as genetics, comorbidities, and lifestyle. Tumor markers derived from metabolomic studies are typically organized into comprehensive panels comprising numerous metabolites, due to the inherent complexity and dynamic nature of metabolic pathways, which rarely allow a single metabolite to fully represent tumor-specific alterations. Nevertheless, even within panels, individual metabolites that enhance the potential to indicate tumor development have been identified in certain cancers. As an example, elevated levels of sarcosine have been associated with prostate cancer [139,140,141], while the phosphorylation and increased concentrations of carnitine-containing metabolites have been linked to breast cancer [114]. Among various tumor-associated metabolic alterations, elevated levels of 2-hydroxyglutarate, identified as an oncometabolite, have been observed in multiple cancer types, including glioma, multiple myeloma, and colorectal cancer [142].

#### 3.1.2. Advancing Cancer Diagnostics Through Multi-Omics Integration

Analyses based on a single omics layer (such as genomics, transcriptomics, or proteomics) are often insufficient to reflect the molecular complexity of cancer. While genomic analyses are valuable for revealing an individual’s genetic predisposition, the functional consequences of such variants often remain unclear [110]. Similarly, a biomarker identified at the proteomic level may result from changes at several molecular layers, including genetic mutations, epigenetic modifications or metabolic derangements [110]. Therefore, by integrating multiple molecular dimensions, multi-omics approaches may provide a holistic framework for analyzing various molecular components and facilitate the identification of disease-specific biomarkers with improved sensitivity and specificity [104]. Although the benefits of multi-omics are increasingly acknowledged, the majority of studies focused on early cancer detection have integrated only two omics layers (such as genomics and epigenomics), falling short of fully leveraging multi-dimensional data [143]. Xu et al. integrated three omics layers, genomics, epigenomics, and transcriptomics, with a machine learning approach to differentiate ductal carcinoma in situ (DCIS) from breast cancer [144]. Since DCIS carries a risk of progression to invasive disease, reliable markers are needed for accurate early diagnosis [145]. In the study, gene expression analysis provided the highest classification accuracy, while the inclusion of DNA methylation and copy number variation data further increased the classification sensitivity. As a result, a 10-gene diagnostic panel was developed to distinguish DCIS from breast cancer with high accuracy, where the AUC was 0.99. The diagnostic potential of the panel was validated by RNA sequencing and transcriptomics using an independent external validation cohort [144].

Many novel biomarkers emerged through multi-omics technologies such as *CDH3* [146] and INSM-1 [147] in thyroid cancer, *DHFR* and *E2F3* [148] in neuroblastoma, and *IQGAP1* [149] in gastric cancer. From a broader perspective, one initiative designed to maximize the benefits of multi-omics approaches is the development of Integrated Personal Omics Profiling (iPOP). This was designed for profiling an individual over time by integrating multi-omics data with clinical evaluations, using wearable devices and advanced molecular profiling, with the goal of developing predictive models for long-term health outcomes [150]. Although iPOP-generated data showed great potential to inform health and disease management [151,152], larger-scale studies are needed to confirm and expand these findings, including possible applications in the field of oncology.

#### 3.1.3. Liquid Biopsy for Omics Analysis

Invasive tissue biopsies, commonly used for omics analyses, are not suited for cancer screening or early diagnostics [5]. The detection of CTCs, or the circulating products of tumors, such as fragments of nucleic acids or proteins in blood samples, provides a non-invasive alternative to conventional tissue biopsies [107]. This approach, referred to as a liquid biopsy, creates an opportunity for the implementation of omics analyses for screening, early cancer detection, and personalized omics profiling [143]. Liquid biopsy enables the non-invasive detection of tumor-specific mutations through the analysis of circulating free DNA (cfDNA). Examples of mutations detected via cfDNA are *EGFR* and *KRAS*, which are clinically important for the management of lung cancer [153,154], and mutations in *TERT* and *TP53*, which have broader applicability across various cancer types [118] (Figure 1B).

Another notable example is the detection of *SEPT9* methylation in cfDNA, which has achieved clinical utility for the early detection of colorectal carcinoma [155]. CTC analysis has also been studied across a wide range of cancer types to date. Gu et al. have provided a comprehensive summary of CTC-associated tumor markers [101]. Recently, an attempt was made to identify a liquid biopsy signature (including CTCs, cfDNA, extracellular vesicles, circulating immune system, cf-nucleosomes, proteins, and microbiota) and generate a multi-marker panel in blood samples from patients with histologically confirmed pancreatic ductal adenocarcinoma to facilitate early detection as the first phase of a non-interventional prospective clinical trial [156]. On the other hand, several studies have presented multiple cancer screening tests based on omics studies of liquid biopsies [157,158,159,160]. These tests utilize various omics technologies, while leveraging traditional imaging and machine learning approaches to increase diagnostic accuracy, and aim to detect all cancer types while confined to their primary tissues [157,158,159,160]. As an example, Cohen et al. developed a liquid biopsy-based multi-cancer screening panel that integrates genetic mutations in cfDNA (across 1933 loci in 16 cancer-associated genes) with serum levels of eight protein biomarkers, including CA 125, CEA, and CA 19-9 [161]. The diagnostic sensitivity ranged from 91% to 98% for four cancer types (ovarian, liver, stomach, and pancreatic) while the specificity exceeded 99%. Notably, the sensitivity of the panel for stage I liver cancer was reported as 100%. The test yielded suboptimal results for breast and lung cancers. Nevertheless, the study highlights the potential utility of similar multi-analyte approaches, especially when optimized with carefully selected mutation targets and protein biomarkers tailored to specific cancer types [161]. Meanwhile, there is an increasing interest in the early diagnosis of lung cancer through liquid biopsies [162,163,164]. Mathios et al. performed genomic fragmentation analysis based on cfDNA analysis from liquid biopsy for the early diagnosis of high-risk lung cancer patients (e.g., with smoking history and pulmonary symptoms) [165]. Combining fragmentation profiles, clinical risk factors, CEA levels, and machine learning approaches, followed by CT imaging, 91% of patients with stage I/II lung cancer were detected with 80% specificity [165]. Moreover, liquid biopsies can be performed using advanced point-of-care biosensor platforms, enabling real-time monitoring and facilitating more dynamic, non-invasive cancer management strategies [166].

#### 3.1.4. Integration of Omics Technologies and Artificial Intelligence

Studies conducted over many years have accumulated extensive data across multiple omics layers. Multi-omics approaches provide a comprehensive molecular landscape by enabling cross-validation across different omics layers. However, the integration of multi-omics data requires dedicated strategies to manage, harmonize, and effectively interpret diverse omics layers [167,168]. Moreover, developing multiplex panels composed of potential biomarkers is a valuable approach to achieving improved diagnostic performance, although it may also require more detailed and complex analyses [167,168]. Consequently, the need to process large-scale datasets and to develop diagnostic panels composed of biomarkers acting in synergy has created a demand for specialized algorithms. In today’s technological era, leveraging a wide range of artificial intelligence and machine learning approaches, such as support vector machines, random forest (RF), and deep neural networks, has become a promising and increasingly adopted strategy [169]. To facilitate multi-omics integration, various pathway and network-based systems, as well as hierarchical biological modules (e.g., deepKEGG), have been developed which leverage biological relationships between different molecular elements such as genes, microRNAs, and proteins [168,170]. On the other hand, the combination of multiple machine learning methods is frequently used as an effective strategy for selecting biomarker panels that enhance diagnostic performance [144,170,171]. As a result, inferential information has been derived from datasets that would likely be incomprehensible through manual analysis alone.

To date, various omics-based studies supported by artificial intelligence have been carried out for diagnostic purposes. These include the identification of novel miRNA signatures related to renal cell carcinoma [172], transcriptomic biomarkers for the diagnosis of NSCLC and HCC [173,174], and epigenomics profiles distinguishing malignant and benign tumors of the central nervous system [175]. Tao et al. developed a machine learning-based statistical model using cfDNA-based genome sequencing to improve the detection accuracy of early-stage HCC. The model, based on an RF algorithm adapted for whole-genome sequencing analysis (a novel weighted RF-driver model), achieved a maximum predictive performance with an AUC of 0.920 in independent validation cohorts [176]. Commercial cancer diagnostic and screening initiatives have also employed machine learning methods and yielded promising results [161,177]. Among these, a blood-based test that integrates cfDNA and protein biomarkers and applies machine learning—including convolutional neural networks—has been evaluated and validated in large-scale clinical trials for early CRC detection [178,179]. Accordingly, the data obtained from the PREEMPT CRC^®^ trial (NCT04369053), which included 32,731 participants across more than 200 centers in an average-risk population, demonstrated a sensitivity of 79.2% for CRC and a specificity of 91.5% for advanced colorectal neoplasia [177].

Recently, Nagarkar et al. developed a multi-cancer early detection test based on the serum metabolome with the aid of machine learning [180]. Through untargeted serum metabolite profiling by high-resolution mass spectrometry, followed by selective filtering steps, a panel of 2709 metabolites was identified. The researchers listed 100 specific metabolites that contributed the most to diagnostic accuracy. When implemented, the panel was able to detect 30 cancer types, including early stages, with a sensitivity of 90–100% and a specificity of 99.2%. For example, the detection sensitivity and specificity for Stage I–II ovarian cancer were reported to be 100% and 96%, respectively [180].

As discussed above, since the early 2000s, significant efforts and extensive research have been undertaken to enable early cancer detection through the use of omics technologies. Numerous candidate tumor biomarkers have been rapidly discovered and reported in preclinical studies; however, translating these biomarkers into clinical applications requires large-scale validation efforts that follow a longer timeline. Many biomarkers reported to exhibit high performance were initially identified in small, single-center studies with limited patient populations, and are often subject to reproducibility limitations. For example, a recent study with approximately 12 years of follow-up investigated the association between 1463 plasma proteins and the risk of 19 different cancers, including CRC [181]. The authors reported that they were unable to replicate previously identified protein–risk associations, particularly those found in small CRC cohorts (n ≤ 100) [182,183]. Different sampling procedures and different analytical methodologies result in inconsistencies among study findings. Overcoming these challenges will require the implementation of standardized study protocols. On the other hand, the increasing volume and complexity of data, along with the growing number of studies and candidate biomarkers, have benefitted considerably from advances in bioinformatics and artificial intelligence, which have facilitated the organization, analysis, and interpretation of multidimensional datasets. The integration of artificial intelligence may provide the necessary support to unlock the long-anticipated potential of advanced omics technologies. However, the technologies and the resulting data are quite heterogeneous, and sophisticated methodologies are still required to properly integrate them and ensure clinical applicability [184]. Ongoing efforts are focused on validating promising single biomarkers, multi-marker panels, and molecular profiles across large patient cohorts. Feasible clinical translation requires reproducibility in independent research groups, standardized detection technologies, accessibility, and validated improvements in both accuracy and cost-effectiveness compared to existing diagnostic practices.

### 3.2. Continuous Monitoring of Malignancies Using Wearable Biosensors for Tumor Markers

Considering cancer’s major global impact on health, monitoring techniques for early cancer diagnosis have consistently attracted significant interest. Although traditional diagnostic methods such as imaging and biopsies are essential in the current state of healthcare technologies for cancer, they have fundamental limitations, including healthcare professional and laboratory resource requirements, the invasiveness of procedures, and high costs. The requirement for patients to visit specialized laboratories for measurements limits accessibility and the feasible testing frequency for screening, early diagnosis, and monitoring—particularly in high-risk individuals and those with rapidly progressing malignancies characterized by short tumor doubling times (Table 1), where more frequent assessments are critical [185]. Therefore, there is a growing demand for innovative technological approaches in the digital era. The development of wearable biosensors based on non-invasive sampling for the continuous measurement of tumor markers may provide effective clinical monitoring in oncology.

In recent years, wearable sensor technologies have been significantly improved and are now actively used in health and disease management [186,187,188]. These devices can analyze various body fluids, including ISF, seminal fluid, sweat, urine, tears, saliva, and breath [189]. Biosensors that are directly placed on the body, such as patches or electronic tattoos, integrated into clothing, or incorporated into accessories like smartwatches, rings, and bandages, collect and analyze personal physiological and activity data [188,189]. The wireless transmission of this data to the smart devices of users and healthcare providers may facilitate remote monitoring. While continuous measurements and real-time health feedback via wearable biosensors have become widespread for the elderly, individuals with disabilities, and chronic disease management, its application in oncology remains limited [190,191]. To the best of our knowledge, wearable biosensors capable of continuously measuring tumor markers in body fluids are not yet available; however, significant progress has been made, leading to the development of fully portable platforms [192] (Table 3).

With today’s advanced technology, tumor marker biosensors can perform rapid and sensitive on-site measurements using miniature devices. In the early stages of tumor development, due to the localized settlement of cancer cells, tumor marker concentrations in blood remain low, while levels in biological fluids such as sweat, tears, and ISF are typically even lower [224,225]. The detection limits of conventional diagnostic techniques are typically too high to detect these biomarkers at such minute levels. However, these can be effectively detected via biosensors. Biosensors employ various measurement technologies, including optical, colorimetric, piezoelectric, and electrochemical methods, which have been comprehensively described in several reviews [226,227]. Conductive materials used in portable biosensors such as MXenes [228], metal-organic frameworks [229], quantum dots [193], and conductive polymers [230] exhibit enhanced sensitivity, thus enabling the identification of tumor markers at otherwise undetectable levels. Biosensors can detect tumor markers, including CEA, AFP, PSA, CA 19-9, and CA 125 at ultra-low concentrations, e.g., pg/mL or even fg/mL [231,232].

Moreover, biosensors enable a rapid sample analysis, i.e., an instantaneous assessment of biomarker levels [233], particularly electrochemical biosensors, which show a response time of less than 5 min for CEA [234] and PSA [235], and even 35 s for AFP [202].

Biosensor technologies based on the sampling of blood and other body fluids are discussed below.

#### 3.2.1. Biosensors for Tumor Biomarker Analysis from Blood

**Figure 2:**
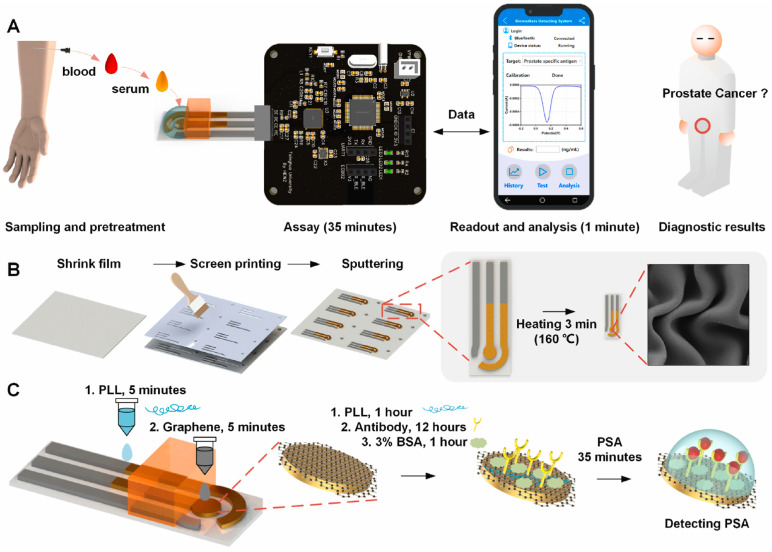
Schematics of the portable biosensor for prostate cancer diagnostics. (**A**) General detection process and smartphone-mediated readout. (**B**) Electrode system and fabrication process. (**C**) Surface modification process and PSA detection. PLL: poly-L-lysinehydrobromide; BSA: bovine serum albumin. (Adapted with permission from Reference [205], published by Elsevier 2023, https://doi.org/10.1016/j.bios.2023.115193.

Since a great majority of clinically validated tumor biomarkers originate from the blood (Table 2), most biosensors are designed for the blood-based samples. Today, biosensors for nearly all common blood tumor markers are available; however, many still require laboratory-based set-ups [227,236]. Advances in miniaturized materials offer the potential for adaptation to point-of-care applications. Currently, portable sensors developed for fully integrated on-site use, and capable of operation with small table-top set-ups, are exemplified in Table 3. Advanced biosensors are now available for various applications, featuring sophisticated operating systems, integrated memory, wireless network connectivity, and the capability to provide real-time feedback to smartphones [237]. He et al. described a polymer-based compact and portable immunosensor for PSA [205] (Figure 2). The electrode surface was supported by gold film for enhanced specificity, and air plasma treatment and graphene were used for increased sensitivity where LOD was 0.38 fg/mL. Results are monitored via a synchronized smartphone, thus facilitating the early diagnosis of prostate cancer. In another study by Ibanez-Redin et al., a flexible electrochemical immunosensor was presented for CA 19–9 measurement (LOD: 0.07 U/mL) in serum and cell lysates by coating screen-printed carbon electrodes with carbon black and polyelectrolytes [197]. This biosensor is suitable for large-scale production and disposable use and offers the potential for frequent and easy CA 19-9 measurements in pancreatic cancer patients.

AFP is routinely measured by immunological assays (Table 2). As an alternative, various portable biosensors were developed for AFP detection [202,203,211,238]. A very recent study introduced an electrochemical immunosensor based on a nanocomposite composed of toluidine blue, gold nanoparticles, Fe_3_O_4_, and reduced graphene oxide (rGO) [202]. This immunosensor can detect serum AFP in just 35 s, demonstrating high concordance with ELISA-measured results in clinical serum samples (R^2^ = 0.998). The system equipped with a simple operating system was proposed for liver cancer screening at the point of care [202]. Chu et al. [211] developed silk-based and flexible sensors for detecting AFP and CEA biomarkers in blood. Photonic crystal barcodes provide excellent color properties and high-sensitivity measurements for fluorescence-based analysis (with a detection range of 10–1000 ng/mL for both AFP and CEA), while the graphene-based microcircuit system also functions as a motion sensor [211]. Researchers have also incorporated multilayered microchannels into biosensors for wearable applications, providing a fluidic pathway for sample transport and detection. Since the sensor currently uses a fingertip blood sample, integrating microneedles into the system may allow its adaptation for wearable use [211].

A recent comprehensive review of Foroozandeh et al. covered analytical features of biosensors for ovarian cancer diagnosis [239]. Among these, Srilikhit et al. developed a point-of-care immunosensor for the simultaneous detection of CA 125 (LOD: 0.6 U/mL) and CEA (LOD: 0.15 ng/mL) based on whole blood sampling [206]. By integrating a plasma separation membrane within a fluidic cell (Flu-iDCE) fabricated from an acrylic sheet, the system enables the direct analysis of whole blood, eliminating the need for centrifugation, thus offering a simplified sample preparation process. This innovation provides greater accessibility and operational convenience for the rapid and efficient detection of CA 125 and CEA [206]. In another example, a screen-printed electrochemical biosensor with multi-walled carbon nanotubes/thionine/gold nanoparticles for the detection of CA 125 transmits the measurement results to smartphones via Bluetooth. The device, which analyzes serum samples, also stands out with its low limit of detection (2 mU/mL) [200].

Biosensors developed for CTCs are also becoming popular in early cancer diagnosis [232]. Various immunosensors have been designed to detect and isolate CTCs, which are present in extremely low concentrations in circulation (see Section 2.5. Circulating Tumor Cells), especially in early stages. Among these, several methods utilizing immunoaffinity and immunomagnetic bead-based approaches, enhanced with micro and nanostructures to improve CTC detection efficiency, have been commercialized [240,241]. However, due to the differences between CTC detection technologies and the lack of a standardized method, diagnostic or prognostic evaluation criteria currently depend on the measurement method [107].

Biosensors based on blood sampling are not entirely suitable for continuous or frequent monitoring. However, for many biosensors that have not yet been standardized for clinical use, using blood as a sample enables their performance to be validated by comparison with conventional diagnostic tests [227].

Alternatively, biomarker measurements based on easily accessible body fluids are considered more feasible approaches for non-invasive, continuous monitoring.

#### 3.2.2. Biosensors for Tumor Biomarker Analysis from Other Body Fluids

Early cancer detection efforts are increasingly focused on non-invasive technologies that enable continuous monitoring. Wearable or portable sensors that can analyze easily accessible body fluids such as sweat, saliva, urine, tears, and ISF offer suitable platforms for more frequent measurements. However, clinical application of these fluids for early diagnosis requires validated cancer biomarkers and standardized measurement methods [242]. Biosensors developed for the detection of tumor markers in various body fluids are summarized below.

**Figure 3:**
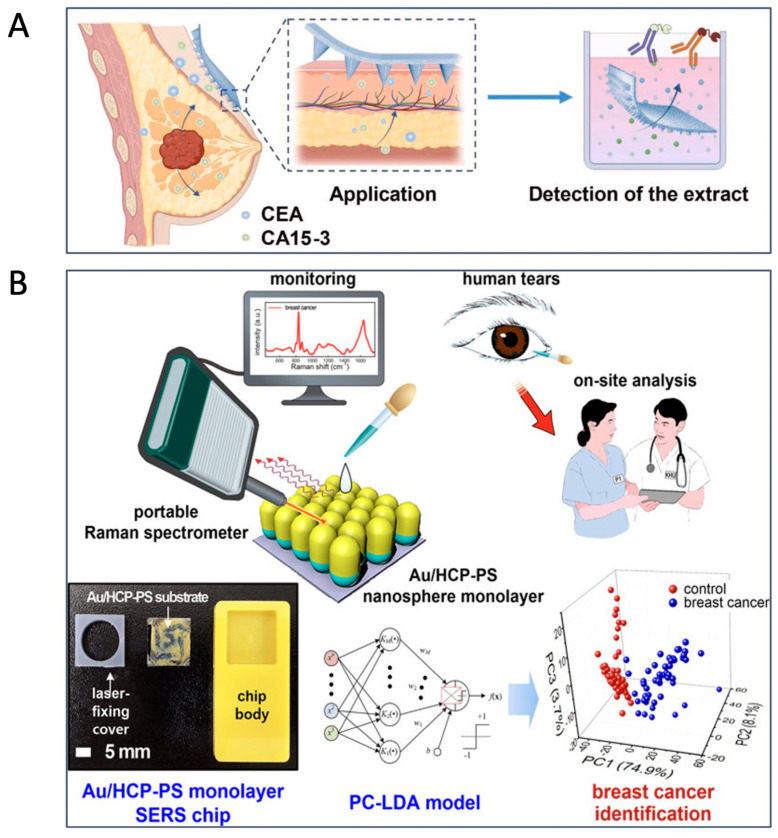
Non-invasive early detection strategies for breast cancer. (**A**) Microneedle patch that enables minimally invasive and rapid removal of interstitial fluid for CEA and CA 15-3 measurements (Adapted with permission from Reference [245], published by Elsevier 2023, https://doi.org/10.1016/j.cej.2023.145036). (**B**) A portable plasmonic SERS platform based on Raman scattering profiles of tear fluid for breast cancer detection. Au/HCP-PS: gold-decorated, hexagonal-close-packed polystyrene; PC-LDA: principal component linear discriminant analysis. (Adapted with permission from Reference [220], copyright (2020) American Chemical Society, https://doi.org/10.1021/acsami.9b19421.

ISF is the body fluid that surrounds tissue cells and has a composition quite similar to blood plasma [243]. Tumor markers such as AFP, CEA, and CA 15-3 can be detected in ISF [244]. For example, ISF collected from breast tissue offers great detection potential for various biomarkers in early stage breast cancer. Huang et al. developed a microneedle patch that enables minimally invasive and rapid extraction of ISF (~1.29 µL ISF in 1 min) from breast tissue. The patch was fabricated by UV-induced polymerization and cross-linking of acrylic acid with gelatin methacrylate, which enables the efficient removal of the sample. This approach offers the potential for earlier breast cancer detection by analyzing tumor markers, CEA and CA 15-3, in ISF, compared to traditional blood tests and imaging methods such as micro-CT and ultrasound (Figure 3A) [245]. Tumor-derived exosomes can also be used as a biomarker for early tumor identification [246]. Park et al. proposed an ISF-based biosensor for measuring glypican-1-positive exosomes for CRC diagnosis. The developed sensor can facilitate early tumor detection by non-invasive means, but additional processing is needed for application in wearable form [247].

Saliva is another easily accessible body fluid enriched in numerous tumor-associated biomarkers. Various biosensors have been developed for the saliva-based measurement of tumor markers, including PSA, CA 125, CA 72-4, CA 19-9, CEA, CYFRA 21-1, p53, TNF-α, IL-1β, and matrix metalloproteinase-9 [248,249,250]. In the study by Bratei et al., 3D needle-type stochastic microsensors were employed for the simultaneous and highly reliable analysis of CA 72-4, CA 19-9, CA 125, and CEA in biological fluids, including saliva. A key advantage of the method is its independence from the complexity of biological samples, ensuring accurate and reliable detection. The innovative sensor design incorporated boron- and nitrogen-decorated graphene, enhancing conductivity and stability [248]. The sensor enabled biomarker analysis with a broad linear range and low detection limits, establishing it as an efficient tool for early cancer screening [248]. Previously, CYFRA 21-1 was proposed as a potential biomarker for oral cancer [250,251]. Various biosensors have been developed for CYFRA 21-1 analysis based on saliva sampling [216,217,252,253]. Among them, Tofighi et al. proposed a portable silver nano-ink-assisted paper immunosensor for enhanced conductivity and stability. Electrochemical measurements provide a wide measurement range (0.0025–10 ng/mL) [216]. In the study, unprocessed real human saliva samples from an early stage oral cancer patient were used; however, the applicability of salivary CYFRA 21-1 as a biomarker for other cancers was not evaluated.

Urine has been used for many years for the detection of various diseases including cancer, due to its non-invasive collection and the presence of a wide range of biological markers.

Biosensors designed for urine analysis generally operate by using small (1 cm) microfluidic/disposable chip-structured sensors [219]. Khan et al. comprehensively listed over 100 urine-based biomarkers associated with various cancers, including lung, breast, colorectal, bladder, and prostate cancer [232]. Among these, biomarkers that can be measured using urinary biosensors are currently limited. For instance, in prostate cancer diagnosis, various sensors have been developed for the detection of volatile organic compounds, potential markers such as glypican-1, annexin A3, and endoglin, as well as tumor-derived DNA and RNA [254]. However, a major limitation remains the lack of validation through large-scale studies. In another example, Yang et al. developed an indium gallium zinc oxide-field effect transistor-based biosensor integrated with a machine learning algorithm for detecting bladder cancer in clinical urine samples [219]. In addition to nuclear matrix protein 22, the analysis of protein biomarkers, carbonic anhydrase 9, cluster of differentiation 47, cytokeratin 18, and cytokeratin 8 was proposed for more accurate bladder cancer diagnosis. The combined analysis of the above-listed five biomarkers achieved a detection accuracy of 95.0%. The system, which transmits analysis results via a wireless Bluetooth unit, was presented as a potential candidate for clinical applications in bladder cancer diagnosis [219].

Sweat, as a body fluid with a rich composition and easy accessibility, has shown a rising trend in biomarker analysis. Numerous wearable biosensors have already been developed for the detection of various biomarkers, including proteins, metabolites, and electrolytes [189]. However, sweat-based measurements of cancer-related biomarkers have not followed a similar trend, and studies in this area remain quite limited. CEA can also be detected in sweat. A wearable electrochemical biosensor was recently developed for rapid and quantitative testing of CEA (with a linear range of 0.2–100 ng/mL). The system employed a microfluidic sweat collector for sampling and AuNPs/rGO-modified screen-printed electrodes for electrochemical analysis [255]. However, additional studies are needed to evaluate its clinical significance.

Tears contain various molecules with the potential to reflect the physiological state of the body [256]. Studies have demonstrated the potential of tears for the early diagnosis and monitoring of various cancers [257,258]. Elevated levels of lactoferrin and cystatin C have been observed in eye cancer, while cystatin SA, lacryglobin, and miRNAs (miR-21 and miR-200c) have been found at higher concentrations in the tears of breast cancer patients [256,259]. Additionally, lacryglobin levels are elevated in the tears of patients with colon, prostate, and lung cancers [257]. An innovative approach involves the use of tear fluid for the early detection of breast cancer through a plasmonic SERS platform equipped with a gold-decorated, hexagonal-close-packed polystyrene (Au/HCP-PS) nanosphere monolayer, enabling femtomole-scale detection [220]. The measurements are based on the Raman scattering profiles of various biomarkers, including collagen, carotenoid, tyrosine, and phenylalanine. This technology reportedly achieved a detection sensitivity of 92% and specificity of 100% for breast cancer [220] (Figure 3B). While tear sampling is non-invasive and can be conducted using absorbent materials or micro-capillary tubes, it presents challenges such as the small sample volume, evaporation issues, and the impact of volumetric changes in tear production on the concentration of its biomolecules.

Exhaled breath contains thousands of distinct organic molecules, and the profile of volatile organic compounds (VOCs) varies between healthy and diseased states [260]. The early and non-invasive detection of lung cancer represents one of the most compelling potentials of breath analysis [261]. Wearable and portable devices designed as electronic noses have attracted significant attention for exhalation-based analysis [260,262]. These devices operate by detecting various VOCs, such as benzaldehyde, 2-ethylhexanol, isopropanol, and n-butanol, which are elevated in lung cancer [263,264]. Recently, Zompanti et al. developed a system in which exhaled breath is collected on absorbent cartridges and subsequently analyzed via a portable device [265]. This system was clinically evaluated to assess changes in the breath-print before and after the surgical resection of lung cancer, and was reported to detect recurrence with 91% accuracy [265]. Portable biosensors able to detect colorectal and prostate cancer from exhaled breath samples have also been developed [266,267]. Since current portable devices for cancer detection are relatively bulky, research is underway to develop miniaturized systems which would be user-friendly, and linked to smartphones [263]. The establishment of standardized protocols holds promise for enabling real-time, non-invasive diagnosis and monitoring of cancer patients.

Recent biosensors have significantly improved by offering a broad linear range, a high sensitivity, and a low detection limit, thus enabling biomarker detection in early stages [232]. Despite the considerable advancement in analytical capabilities [268], the integration of biosensors for tumor marker detection into fully integrated wearable platforms currently lags considerably behind other existing wearable sensor applications.

For instance, non-invasive wearable glucose sensors based on ISF sampling are in use for diabetes management [269]. In addition, researchers have developed a variety of wearable biosensors, including a ring-format device for the sweat-based analysis of female reproductive hormones [270], patch-format sensors for levodopa monitoring in Parkinson’s disease management [271,272,273], sweat-based metabolite detection for dietary applications [274], and also an ultrasound breast patch that provides deep tissue scanning for breast cancer monitoring [275]. Numerous modifications of biosensor technology could be described, reflecting the expanding scope and capabilities of wearable biosensors [189]. Considering the advanced biosensors developed for tumor marker measurement, the integration of non-invasive sampling modules—such as microfluidic technologies—and the use of biocompatible materials that provide sufficient softness and flexibility for on-skin applications would enable the transition of these biosensors into wearable platforms suitable for continuous monitoring.

Biosensor applications still face technical challenges such as ensuring stability, standardization, and reproducibility, as well as dealing with rapid degradation, cross-reactivity, and non-specific interference. The complexity of biological samples can affect detection performance, and structural similarity between molecules may cause false positives. Reliable performance requires strategies that reduce non-specific measurements [276]. Additionally, the detection of most cancer biomarkers requires high-affinity sensors, which is often achieved by aptamers and antibodies [232,239]. Biosensors utilizing natural biological recognition elements often exhibit limited functional stability during continuous use and are sensitive to environmental factors such as temperature fluctuations, which may compromise their long-term performance. However, as an alternative to natural recognition elements, molecularly imprinted polymers, which are resistant to degradation and have long-term stability, have the potential to overcome this limitation by enabling highly selective continuous measurements [223,274,277,278]. In addition, achieving consistent and comparable results with high reproducibility can be challenging across different biosensor platforms developed through various manufacturing processes. To address this, it is important to establish standardized fabrication protocols, measurement procedures, and validated performance criteria to ensure cross-platform compatibility and promote broader clinical and commercial adoption [276].

Nonetheless, elevated levels of tumor markers in non-malignant conditions remain key challenges, leading to diagnostic uncertainty. Advances in wearable biosensor technologies that allow the continuous measurement of tumor markers, along with other developments involving the identification and personalization of cancer-specific tumor markers, are likely to facilitate early stage cancer diagnosis.

### 3.3. Personalization of Tumor Markers

The interpretation of laboratory data, including tumor markers, relies on a comparison with the reference data, so the latter must be reliable. Despite considerable variance across different individuals having the same tumor, common reference intervals (RIs) are still widely used to interpret tumor marker levels. It should be noted that RIs are derived from population data and reflect population-level characteristics rather than individual-specific values [279,280]. Consequently, interpreting levels of tumor markers relying on the population-derived RIs may lead to misinterpretation. Hence, a result considered normal at the population level may, in fact, be abnormal for an individual, and vice versa.

The concentration of biomolecules, including tumor markers, fluctuates around a set point [281]. The range of this fluctuation can be determined using longitudinal data from a group of individuals referred to as within-subject biological variation (CV_I_), which reflects population-level estimates. Alternatively, we can use the individual’s own data, known as within-person biological variation (CV_P_), which is specific to that individual. Finally, the variation in the analyte levels among different individuals is known as between-subject biological variation (CV_G_) [282]. The ratio of CV_I_ to CV_G_ is known as the index of individuality (II). For a biomarker with an II less than 0.6, marked individuality is assumed, and population-based reference intervals (popRIs) are not recommended for interpreting patient results. Conversely, if the II is greater than 1.4, population-based reference intervals are considered appropriate for interpreting measurement results [283]. As shown in Table 1, the majority of tumor markers used in clinical practice exhibit marked individuality, indicating that although popRIs are currently used for interpretation, they are not suitable for these markers. Therefore, it is essential to develop new algorithms to estimate person-specific reference intervals—i.e., personalized reference intervals (prRIs)—as well as algorithms to monitor patients based on their own data rather than population means.

#### 3.3.1. Personalized Reference Intervals for Tumor Markers: A Precision Medicine Approach

An analyte RI has upper and lower limits, thus representing the fluctuation of that analytes around a homeostatic set point (HSP). A prRI is estimated using an individual’s own data. Metabolically, the fluctuation of an analyte corresponds to its CV_P_. Both the HSP and the variation around it can be calculated using serial measurements of analyte concentrations in samples taken at the same time of day over days, weeks, or months, as detailed below.

(1) $$HSP=\frac{x_{1}+x_{2}+x_{3}+\cdots +x_{n}}{n}$$

where *x_i_* is the result of the *i*th measurements and *n* is the number of samples included in the calculation.

The variation around the HSP is the Gaussian combination of the CV_*P*_ and the variation introduced by the measurement procedure, which can be considered as analytical variation (CV_A_), as formulated below:

(2) $$TV=t_{\alpha /2}\times \sqrt{{CV}_{P}^{2}+{CV}_{A}^{2}}$$

where *t_α_*_/2_ is the *t*-distribution value corresponding to *n* − 1 degrees of freedom.

The prRI can be considered as the prediction interval for the next measurement and can be calculated as shown below [284,285,286,287].

(3) $$prRI=HSP\mp t_{\alpha /2}\times \sqrt{\frac{n+1}{n}{(CV}_{P}^{2}+{CV}_{A}^{2})}$$

The use of prediction intervals is described in more detail in the literature, e.g., [288,289,290].

When calculating the prRI value, the number of serial measurements is crucial; the higher the n, the more certain and reliable is the value [287]. Therefore, in principle, n should be ≥5. When this is not feasible, the CV_I_ of the analyte available from the European Federation of Clinical Chemistry and Laboratory Medicine (EFLM) Database should be used instead of the CV_P_ value [291].

In the clinical context, RIs are used to distinguish healthy individuals, and corresponds to the specificity of the algorithm used to estimate the RIs. Specificity can be calculated from the following formula:

(4) $$Specificity=\frac{TN}{TN+FP}$$

where *TN* is the number of true negatives, and *FP* is the number of false positives.

**Figure 4:**
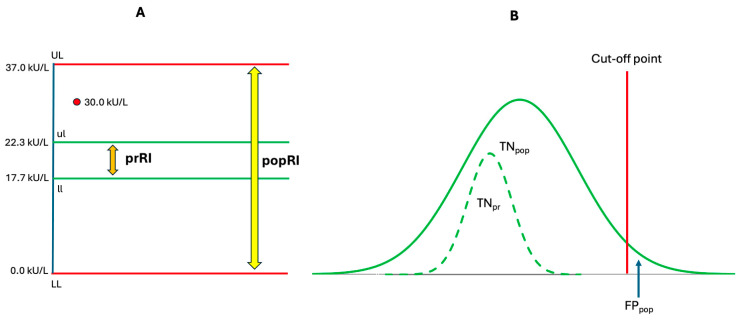
Population-based and personalized reference intervals for tumor markers. (**A**) The popRI and prRI for CA 19-9 are shown as an example. Reference intervals can differ substantially between the population and the individual. As illustrated in the figure, if the individual’s measurement result is 30 kU/L, it would be considered normal when interpreted using the popRI but classified as abnormal when using the prRI. (**B**) Personalized reference intervals (prRIs) and population-based reference intervals (popRIs) represent different levels of tumor marker interpretation. For a given tumor marker, the popRI often does not reflect the individual’s physiological baseline, resulting in potentially low specificity when applied at the individual level. In contrast, prRIs enhance the specificity of reference intervals, allowing for more accurate distinction between healthy and pathological states in individual patients. (**B**): Reprinted with permission from Reference [293], published by MDPI AG 2024, https://doi.org/10.3390/diagnostics14192135.

As shown in Figure 4A,B, the specificity of popRIs is lower than that of prRIs, indicating that the personalization of reference data for a tumor marker can enhance the specificity of its measurement, thus improving its clinical utility. A typical example is CA 19-9, commonly used for the management of colorectal and pancreatic cancer, the latter being one of the most aggressive tumors in humans. The CV_I_ and CV_G_ for CA 19-9 are 4.3% and 57.4%, respectively [291]. Consequently, the II for CA 19-9 is calculated as 4.3/57.4, which equals 0.075. The popRI for CA 19-9 is <37 kU/L [292]. A prRI, calculated for an individual using the CV_I_ and an analytical CV (CV_A_) accepted as desirable (typically equal to half the CV_I_) and a hypothetical set point such as 20 kU/L obtained from 10 serial measurements, shows that the prRI range for this individual (17.7–22.3 kU/L) is significantly narrower than the popRI for CA 19-9 (0–37 kU/L) (Figure 4A). It should be noted that the range of the prRI in this case (22.3–17.7, corresponding to 4.6 kU/L) is narrower than the popRI range (37 kU/L) (Figure 4A). Due to the wide range of CV_G_, the popRI for CA 19-9 is broader than the prRI. Therefore, if this individual’s CA 19-9 level is 30 kU/L, it would be considered normal based on the popRI, whereas for this individual, it is actually elevated and should not be interpreted as normal. Using unsuitable RIs is analogous to following an inaccurate map and can reduce the accuracy of clinical decision-making.

The HSP may vary among individuals, reflecting the population heterogeneity. Therefore, prRIs for tumor markers reflect individual variation and differ significantly from the fixed popRI values commonly used in routine clinical practice. This example illustrates why the specificity of tumor markers is often suboptimal and why they are not commonly employed for the diagnosis of malignant conditions—the reliance on popRIs limits their diagnostic utility. However, if tumor markers were personalized, they could offer significant potential for the early diagnosis of malignancies with high specificity and sensitivity, as detailed below.

#### 3.3.2. Personalized Decision Limits for Tumor Markers in the Diagnosis of Malignant Diseases

**Figure 5:**
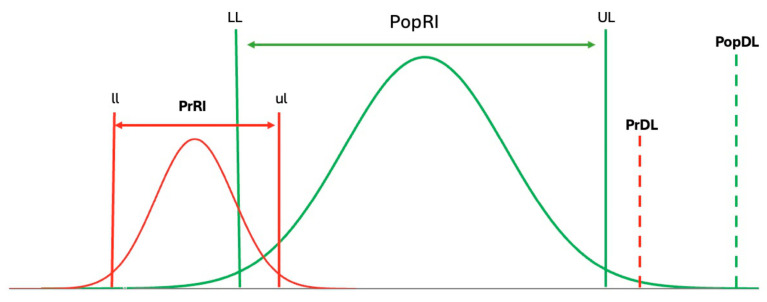
Decision limits are critical thresholds used to diagnose diseases based on biomarker levels. Currently, these limits are typically derived from population-based data. Many tumor markers exhibit marked individuality, a strong need for personalization. Since personalized reference intervals (prRIs) and population-based reference intervals (popRIs) differ significantly for the same tumor marker, the corresponding decision limits may also vary. This discrepancy highlights the need to personalize decision limits to improve diagnostic accuracy at the individual level. UL: upper limit; LL: lower limit; popDL: population-based decision limit; and PrDL: personalized decision limit.

Although RIs are widely used in clinical practice, they are primarily intended to distinguish healthy individuals rather than to diagnose diseases. Therefore, RIs may not be suitable tools for disease diagnosis, which is instead based on decision limits (DL). The estimation of DLs is more complex than the estimation of RIs [294]. Since RIs are estimated using measurement results from healthy individuals, the estimation of DLs requires patient data, and the diagnoses of these patients must be confirmed by other techniques or parameters such as clinical findings, radiological imaging, and pathological examinations. Therefore, the selection of patients to be included in DL studies requires special attention and must be supported by well-documented clinical, radiological, pathological, and other relevant findings. Furthermore, and most importantly, while there is typically a single RI for a tumor marker, there may be multiple DLs. This makes estimating DLs more complex, requiring detailed evaluation for each tumor marker and for each particular clinical condition in which the marker is intended to be used. For example, PSA levels can be elevated in benign prostatic hyperplasia (BPH), prostate cancer, and other conditions such as prostatic inflammation or infections. However, it is well established that PSA elevations tend to be mild in BPH, whereas significantly higher levels are typically observed in prostate cancer. There is a diagnostic gray zone between a healthy prostate and one affected by malignancy, and the DLs used for diagnosing prostate cancer are often well above the upper reference limit of the RI. This concept is illustrated in Figure 5.

Although theoretically possible, the estimation of DLs for individuals is challenging in practice due to the complexity involved in defining DLs for disease diagnosis. However, this does not imply that DLs cannot be personalized. In fact, they can be individualized through an indirect approach, such as simulation studies utilizing popRIs, population-based decision limits (popDLs), and prRIs, provided that the DLs for tumor markers are well established for specific cancer types [25]. Furthermore, a direct relationship must exist between the presence of the tumor and the concentration of the corresponding tumor marker in the blood.

The difference between the upper limit of the popRI and the popDL represents the critical difference, illustrating the minimum concentration gap of tumor markers between healthy individuals and those diagnosed with cancer (Figure 5). A similar percentage change can be expected at the individual level, as formulated below [189].

(5) $${popRC}_{DL}=\frac{L_{popRI}-popDL}{L_{popRI}}$$

Here, *popRC_DL_* represents the population-based relative change from the clinically significant limits of the RI to the popDLs, while *L_popRI_* denotes the clinically significant limit of the popRI.

(6) $$prDL=L_{prRI}\mp L_{prRI}\times {popRC}_{DL}$$

The specificity of an analyte pertains to its ability to correctly identify healthy individuals and is therefore associated with RIs, whereas sensitivity refers to its ability to correctly identify diseased individuals and is related to the DLs of the analyte used for disease diagnosis, as formulated below.

(7) $$Sensitivity=\frac{TP}{TP+FN}$$

where *TP* corresponds to true positives and *FN* corresponds to false negatives identified by the DLs.

As shown in Figure 5, prDLs have great potential to increase the sensitivity of tumor markers and, consequently, enhance their clinical significance in the diagnosis of malignant diseases.

#### 3.3.3. Personalized Reference Change Value for Monitoring Disease Progression and Treatment Response

RIs can be used to distinguish healthy individuals based on analyte levels; however, monitoring individuals for disease progression, evaluating treatment effectiveness, and assessing treatment side effects cannot rely solely on RIs. Therefore, a new algorithm is needed for the objective evaluation of individual monitoring in both healthy and diseased conditions. The monitoring of individuals can be performed using serial measurements of analytes during the course of disease. The significance of changes in analyte concentrations can be assessed using the reference change value (RCV), which is calculated as shown below.

(8) $${RCV}_{P}=t_{\alpha /2}\times \sqrt{2}\times \sqrt{{CV}_{A}^{2}+{CV}_{P}^{2}}$$

**Figure 6:**
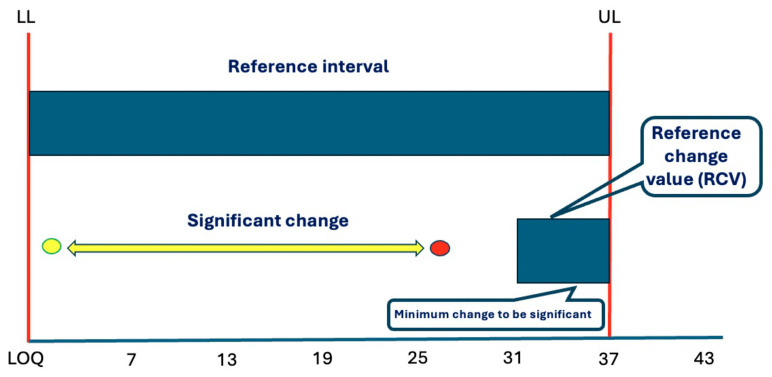
The reference change value (RCV) indicates the magnitude of significant change between serial measurements and is a critical parameter in disease monitoring using biomarkers. Although sequential results may fall within the reference interval and be considered normal by conventional approaches, they can, in fact, differ significantly from each other. The RCV helps to identify such clinically meaningful changes, even when values remain within the population-based reference interval. The yellow and red circles represent the first and second measurement results of the same tumor markers in samples taken at different time points.

If the difference between two measurements obtained from different samples taken at the same time on different days, weeks, or months (i.e., the delta value) is lower than the RCV, then this difference can be considered insignificant at a specified probability level, such as 95% [281,295]. It indicates that this difference can be attributed to inherent variation arising from the measurement procedure and biological variation, and is therefore likely to be insignificant. Conversely, if the delta value exceeds the RCV, the difference is considered significant at a given probability level (e.g., 95%) and cannot be explained solely by analytical and/or biological variation. In such cases, the observed difference should be interpreted as resulting from other factors, such as disease progression, treatment effect, or drug-related side effects (Figure 6).

## 4. Conclusions

Although tumor markers are currently used primarily for monitoring malignant diseases, they possess significant potential in the broader management of cancer. More than a century of experience has shown that relying on a single biomarker is often insufficient for the early and accurate diagnosis of malignancies due to limitations in sensitivity and specificity. Therefore, combining multiple tumor markers provides a more robust diagnostic strategy. This insight calls for a new paradigm in the use of tumor markers—one that redefines their role in the early detection, monitoring, and personalized treatment of malignant diseases.

Emerging technologies such as omics platforms offer powerful avenues for the discovery of novel biomarkers for malignant diseases, while wearable biosensors enable the continuous, real-time monitoring of individuals. Given the biological heterogeneity of tumors, personalizing tumor marker interpretation becomes essential. Furthermore, artificial intelligence can integrate personal health data with wearable biosensor outputs to develop individualized algorithms for the early detection and effective management of malignancies. Together, these innovations promise to transform tumor marker applications and significantly advance personalized oncology.

## Figures and Tables

**Table 1:** Tumor volume doubling times in different cancers, key tumor markers, and their index of individuality.

Cancer	Subtype	Tumor Volume Doubling Time	Tumor Marker	II	Ref.
Lymphoid neoplasms	Burkitt’s lymphoma	24–48 h	LDH	0.37 *	[10]
Testicular cancer	Non-seminoma	21 days ^†^	AFP	0.08 *	[11]
Brain tumor	Glioblastoma	29.8 days ^‡^	VEGF	-	[12]
Lung cancer	Small cell lung cancer	73 days * ^¤^	NSE	0.66 *	[13]
			proGRP	0.29	
Ovarian cancer		90 days ^‡^	CA 125	0.34 *	[14]
Lung cancer	Squamous cell lung cancer	140 days * ^¤^	SCC	-	[13]
Liver cancer	Hepatocellular carcinoma	140 days * ^¤^	AFP	0.08 *	[15]
Pancreatic cancer		144 days ^‡^	CA 19-9	0.07 *	[16]
Breast cancer		180 days * ^†^	CA 15-3	0.12	[17]
Gastric cancer		186 days (T1) ^†^	CEA	0.11 *	[18]
			CA 19-9	0.07 *	
Colorectal cancer		211 days ^‡^	CEA	0.11 *	[19]
Lung cancer	Adenocarcinoma	223 days * ^¤^	CEA	0.11 *	[13]
			CYFRA 21.1	0.67	
Thyroid cancer	Medullary thyroid carcinoma	1.6 years ^†^	Calcitonin	0.2 *	[20]
			Thyroglobulin	0.14 *	
Prostate cancer		>2 year (89% of patients)	PSA	0.16 *	[21,22]
Thyroid cancer	Papillary thyroid carcinoma	>5 year (71.8% of patients)	Thyroglobulin	0.14 *	[23]

II: The index of individuality was calculated using data provided in the EFLM database. * The data presented are based on systematic review and/or meta-analysis. ^¤^ Pooled mean, ^†^ mean, and ^‡^ median.

**Table 2:** Overview of key tumor markers: measurement methods, clinical applications, and year of investigation.

Tumor Markers	Method	Primary Malignancy	Other Malignancies	Non-Malignant Conditions	Year	Ref.
Bence Jones Protein	IFE, SFLC	Multiple myeloma *	Non-Hodgkin’s lymphoma, Waldenström’s macroglobulinemia	Pre-malignant plasma cell disorders	1847	[27]
hCG	ECLIA, CLIA	Germ cell and testicular tumors, gestastional trophoblastic neoplasia *	Lung cancer	Hyperthyroidism, chronic renal failure	1956	[28]
AFP	ECLIA, CLIA	Hepatocellular carcinoma *, germ cell tumors *	Gastric, colorectal, bilary, pancreatic, and lung cancer	Liver regeneration, viral hepatitis, pregnancy	1963	[29]
CEA	ECLIA, CLIA	Colorectal cancer	Breast, lung, gastric, pancreatic, bladder, cervical, thyroid, and hepatic cancers, lymphoma and melanoma	Ulcerative pancreatitis, cirrhosis, colitis, hypothyroidism, Crohn’s disease, COPD	1965	[30]
NSE	ECLIA, TRACE	Neuroendocrine tumors (neuroblastoma, small cell lung cancer)	Medullary thyroid carcinoma, melanoma, pancreatic endocrine tumors	Tuberculosis, COPD, alveolar proteinosis, acute respiratory distress syndrome, silicosis, neurological deficits, ischemia reperfusion, brain injury	1965	[31]
Chromo-granin A	TRACE	Neuroendocrine tumors	Presence of neuroendocrine cells in non-endocrine tumors	Atrophic gastritis, chronic renal injury, chronic heart failure, hypertension, rheumatoid arthritis	1967	[32]
Calcitonin	ECLIA, ICMA	Medullary thyroid carcinoma *	Lung, breast, kidney, and liver cancer	Pulmonary disease, pancreatitis, hyperparathyroidis, pernicious anemia	1968	[33]
Thyro-globulin	LC-MS/MS	Thyroid cancer	None	Graves’ disease, Hashimoto’s disease, and thyroiditis	1975	[34]
SCCA	TRACE	Squamous cell carcinoma (cervical, lung, skin, head and neck)	Esophageal adenocarcinoma, hepatocellular carcinoma	Inverted papilloma, non-malignant pulmonary disease, chronic hepatitis, atopic dermatitis	1977	[35]
PSA	ECLIA, CLIA	Prostate cancer *	None	Urinary tract infections, prostatisis, benign prostatic hyperplasia	1979	[36]
CA 19-9	ECLIA, CLIA	Pancreatic cancer *	Colorectal, biliary tract, liver, gastric, and lung cancer, cholangiocarcinoma, mesothelioma	Liver damage, bile duct obstruction and inflammation, pancreatitis, interstitial pulmonary disease, pulmonary fibrosis, collagen vascular diseases, hypothyroidism, gastric ulcer	1979	[37]
CA 125	ECLIA	Ovarian cancer *	Breast, endometrial, cervix, peritoneal, uterus, lung, and pancreatic cancer, non-Hodgkin lymphoma, hepatocellular carcinoma	Idiopathic pulmonary fibrosis, ovarian cyst, endometriosis, adenomyosis, pelvic inflammation, uterine fibroids, rheumatoid arthritis-related interstitial lung disease	1981	[38]
CA 15-3	ECLIA	Breast cancer	Pancreatic, lung, ovarian, colorectal, and liver cancer	Benign liver and breast diseases	1984	[39]
Inhibin A Inhibin B	ICMA ELISA	Ovarian granulosa cell, mucinous epithelial ovarian and testicular tumors	Endometrial carcinoma, adrenal tumors	Preeclampsia, ovarian cysts	1989	[40]
HE4	ECLIA	Ovarian cancer	Lung cancer, pulmonary adenocarcinoma	Chronic kidney disease, renal failure, kidney fibrosis	1991	[41]
Cyfra 21.1	ECLIA	Lung cancer	Breast, bladder, and pancreatic cancer, hepatocellular carcinoma	Renal failure, liver cirrhosis, benign lung diseases	1993	[42]

COPD: chronic obstructive pulmonary disease, ECLIA: electrochemiluminescence immunoassay; CLIA: chemiluminescent immunoassay; LC-MS/MS: liquid chromatography tandem mass spectrometry; ICMA: immunochemiluminometric assay; TRACE: time-resolved amplified cryptate emission cryptate emission; IFE; immunofixation electrophoresis; and SFLC: serum free light chain. * Current recommended clinical applications involve diagnosis.

**Table 3:** Biosensors used in portable devices for measuring tumor markers in various body fluids.

Biofluid	Biomarker	Method	Detection Limit	Assay Time	Measurement Procedures	Ref.
Serum	CEA	Optical (fluorescence quenching)	6.7 pg/mL	80 min	Paper-based device with mesoporous silica NP and quantum dot signal generation via glucose-triggered fluorescence quenching.	[193]
	CEA	Photoelectrochemical	11.3 pg/mL	~35 min	Paper-based immunoassay platform integrating shell–shell structured photoactive materials.	[194]
	CEA	Optical (scanned image analysis)	0.45 ng/mL	15 min	Lateral flow strip with Au-NP probes and nitrocellulose membranes. Office-type scanner used for quantification.	[195]
	CEA	Optical (SERS)	0.36 pg/mL	~30 min	Pump-free microfluidic chip using a Au-NP-modified SiO_2_ microsphere.	[196]
	SCCA		0.45 pg/mL			
	CA 19-9	Electrochemical (DPV)	0.07 U/mL	~25 min	Flexible SP carbon electrode modified with carbon black-polyelectrolyte multilayer films.	[197]
	CA 19-9	Optical	30 U/mL	35 min	Lateral flow sensor integrating magnetized CNT for low-cost, visual detection on disposable strips.	[198]
	CA 125	Optical (colorimetric)	5.21 U/mL	20 min	Lateral flow platform utilizing Au nanozyme-labeled probes for low-cost, home-usable, and visually quantitative detection.	[199]
	CA 125	Electrochemical (DPV)	2 mU/mL	~25 min	Smartphone-integrated system combining a miniaturized detector and SP electrodes modified with CNT and Au-NP.	[200]
	CA 15-3	Electrochemical, (SWV)	0.95 U/mL	~30 min	Disposable chip device based on NP-modified SP electrodes.	[201]
	AFP	Electrochemical, (SWV)	0.03 ng/mL	35 s	POC biosensor integrating multi-functionalized graphene nanocomposites for rapid biomarker detection.	[202]
	AFP	Photoelectrochemical	74.8 pg mL	1.5 h	Biosensor combining oxygen-doped semiconductor photoelectrodes with digital multimeter readout for simple and low-cost biomarker analysis.	[203]
	AFP	Optical	1.27 ng/mL	2 h	Droplet evaporation-based biosensor utilizing surfactant-modified patterns on plastic substrates for simple, label-free detection.	[204]
	PSA	Electrochemical (DPV)	0.38 fg/mL	20 min	Miniaturized sensor integrating shrink polymer-based electrodes with smartphone-controlled operation.	[205]
	LDH	Optical (colorimetric)	70 pg mL	50 min	Electrophoretic lateral flow sensor integrating battery-powered microfluidics, Au-NP signal transduction, and smartphone-based signal quantification. Small benchtop set-up needed.	[206]
	LDH	Optical (colorimetric)	86 ng/mL (LOQ)	10 min	Smartphone-based lateral flow biosensor using carbon NP for visual detection on disposable strips.	[207]
	Thyroglobulin	Optical (LSPR)	93.11 fg/mL	10 min	Fiber optic localized surface plasmon resonance biosensor integrating Au-NP-coated fibers within a microfluidic channel for simplified detection.	[208]
	miRNA 21	Electrical (direct current)	0.0028 fM	1.5 h	Self-powered platform employing graphdiyne-modified electrodes, physical signal amplification, and smartphone readout.	[209]
Whole blood	CEA	Electrochemical (linear sweep voltammetry)	0.15 ng/mL	~25 min	Fluidic-integrated dual carbon electrode platform fabricated by stencil printing.	[210]
	CA 125		0.6 U/mL			
	CEA	Optical (fluorescence)	10 ng/mL	~10 min	Microfluidic silk patch fabricated by 3D printing for flexible sensing.	[211]
	AFP		10 ng/mL			
	PSA	Optical (fluorescence)	0.08 ng/mL	13–22 min	Power-free and flexible, fluoropolymer microcapillary film device integrated with a smartphone.	[212]
	NSE	EIS	1.15 ng/mL	5 min	Disposable chip-like device, enabling simplified detection using mouse model samples without clinical validation.	[213]
Saliva	CEA	Optical (fluorescence)	0.012 ng/mL	~5 min	Fully integrated platform combining acoustic enrichment and smartphone-based visual detection for easy home monitoring.	[214]
	CEA	Optical (time-resolved photoluminescence)	1.47 pg/mL	10 min	Lab-in-syringe platform integrating lanthanide nanoprobes with dissolution-enhanced luminescence for easy on-site detection.	[215]
	CYFRA 21-1	Electrochemical (DPV, chronoamperometry)	0.025 ng/mL (LLOQ)	4 h	Paper-based platform with silver nano-ink printed electrodes.	[216]
	CEA	Electronic (direct current measurement)	0.148 pg/mL	1 h	Label-free biosensor integrating rGO/melamine-modified electrodes with wired electronic readout system.	[217]
	CYFRA 21-1		0.04 pg/mL			
	CA 15-3	Electrochemical (DPV)	0.56 U/mL	1 h	Immunosensor integrating SP paper electrodes modified with AuNPs for simple detection on disposable platforms.	[218]
Urine	NMP22, CA9, CD47, CK8, CK18	Electrical (FET)	≪pg/mL	~5 min	IGZO FET-based urinalysis device integrated with wireless data transfer and smartphone interface for the simultaneous detection of five bladder cancer markers.	[219]
Tears	Raman spectral profile	Optical (SERS)	100 fM	~5 min	Label-free Au/HCP-PS biosensor combined with a hand-held Raman spectrometer enables the detection of breast cancer with 96% classification accuracy.	[220]
Artificial sample	NSE	EIS	1.005 ng/mL	5 min	Microfluidic chip incorporating Au-modified electrodes for simplified detection, without clinical validation.	[221]
	CEA	Optical (fluorescence)	3.1 ng/mL	20 min	Microfluidic device combining magnetic single-bead trapping with acoustic micro-mixing. Small benchtop set-up needed.	[222]
	PSA		0.028 ng/mL			
	CA 15-3	Electrochemical (SWV)	0.909 mU/mL	20 min	Disposable sensor platform utilizing MIPs as alternative to natural sensing elements for stable and selective detection.	[223]

SERS: surface-enhanced Raman scattering; SCCA: squamous cell carcinoma antigen; si: silica; DPV: differential pulse voltammetry; CNT: carbon nanotube; SWV: square wave voltammetry; POC: point of care; Au/HCP-PS: gold-decorated/hexagonal-close-packed polystyrene; LSPR: localized surface plasmon resonance, rGO: reduced graphene oxide; FET: field-effect transistor; SP: screen printed; EIS: electrochemical impedance spectroscopy.

## Data Availability

No new data were created or analyzed in this study. Data sharing is not applicable to this article.

## Supplementary Materials

The following supporting information can be downloaded at https://www.mdpi.com/article/10.3390/biom15071011/s1. Novel Tumor Markers; Table S1. Genetic alterations; Table S2. Tissue markers; Table S3. Serum markers; Table S4. Urinary markers.
