# Supplementary material for: The Future of Tumor Markers: Advancing Early Malignancy Detection Through Omics Technologies, Continuous Monitoring, and Personalized Reference Intervals
Source: Biomolecules. 2025 Jul 14;15(7):1011. doi: 10.3390/biom15071011 (PMC12294060; doi:10.3390/biom15071011)
Supplement: Supplementary file 1 [file biomolecules-15-01011-s001.zip › biomolecules-3660587-supplementary.pdf]

The Future of Tumor Markers: Advancing Early Malignancy Detection through Omics Technologies, Continuous Monitoring, and Personalized Reference Intervals

SUPPLEMENTARY MATERIAL

Novel tumor markers

Table S1. Genetic Alterations

Tumor Marker		Associated Cancer	Ref.
BARD1	BRCA1-associated ring domain	Breast Cancer	[1,2]
CDK12	Cyclin-dependent kinase 12	Breast Cancer	[3]
CDKN2A	Cyclin-dependent kinase inhibitor 2A	Melanoma	[4,5]
CHEK2	Checkpoint kinase 2	Breast Cancer	[6]
CTNNB1	Catenin Beta-1	Hepatocellular Carcinoma	[7,8]
HOXB	Homeobox B genes	Prostate Cancer	[9]
LIN28B	Lin-28 homolog B	Neuroblastoma	[10]
NRAS	Neuroblastoma Rat Sarcoma Viral Oncogene Homolog	Multiple Myeloma	[11]
PALB2	Partner and localizer of BRCA2	Breast Cancer	[12]
PTPN11	Protein tyrosine phosphatase nonreceptor type 11	Neuroblastoma	[13,14]
RAD51	RAD51 recombinase	Breast Cancer	[15]
SEPT9	Septin 9	Colorectal Cancer	[16]
SMAD4	SMAD family member 4	Pancreatic Cancer	[17]
SPI1	SPI-1 proto-oncogene	Neuroblastoma	[18]
TERT	Telomerase Reverse Transcriptase	Hepatocellular Carcinoma	[7]
TWIST1	Twist family transcription factor 1	Bladder Cancer	[19]
WASCH5	WASH complex subunit 5	Gastric Cancer	[20]

Table S2. Tissue Markers

Tumor Marker		Associated Cancer	Ref.
ACTBL2	Actin beta-like 2	Colorectal cancer	[21]
ADGRG1	Adhesion G protein-coupled receptor G1	Ovarian cancer	[22]

AMIGO2	Adhesion Molecule with Ig Like Domain family 2	Prostate cancer	[23]
C1QB	Complement C1q B-Chain	Gastric cancer	[24]
CDH3	Cadherin 3	Thyroid Cancer	[25]
CDK 3/5/8	Cyclin-dependent kinase 3/5/7	Colorectal cancer	[26]
CSPG4	Chondroitin sulfate proteoglycan 4	Melanoma	[27]
DHFR	Dihydrofolate Reductase	Neuroblastoma	[28]
E2F3	E2F Transcription Factor 3	Neuroblastoma	[28]
EPCAM	Epithelial cell adhesion molecule	Ovarian cancer	[22]
ESRP1	Epithelial splicing regulatory protein 1	Ovarian cancer	[22]
GARS1	Glycyl-tRNA synthetase	Bladder cancer	[29]
INSM-1	Insulinoma-associated protein 1	Thyroid Cancer	[30,31]
IQGAP1	GTPase Activating Protein-1	Gastric cancer	[32]
MCM	Minichromosome Maintenance Proteins	Bladder cancer	[33]
PRC1	Protein regulator of cytokinesis-1	Hepatocellular carcinoma	[34]
SATB2	Special AT-Rich Sequence-Binding Protein 2 ,	Colorectal cancer	[35]
TYROBP	TYRO protein tyrosine kinase binding protein,	Gastric cancer	[24]

Table S3. Serum Markers

Tumor Marker		Associated Cancer	Ref.
1-MA	1-Methyladenosine	Prostate cancer	[36]
Acetylcarnitine		Hepatocellular Carcinoma	[37]
AGO2	Argonaute-2	Multiple myeloma	[38]
AGT	Angiotensinogen	Gastric cancer	[39]
Anxa2	Annexin A2	Ovarian cancer Hepatocellular carcinoma	[40,41]
APO-A2	Apolipoprotein A2	Breast Cancer, Hepatocellular Carcinoma	[42,43]
Apo-AIV	Apolipoprotein A-4	Prostate Cancer	[36]
Benzoic acid		Colorectal cancer	[44]
C163A		Lung cancer	[45]
C3fc	C3f complement	Colorectal cancer	[46]

C4BPA	C4b-binding protein α-chain	Pancreatic cancer	[47]
Dermcidin		Melanoma	[48]
DKK1	Dickkopf-1	Hepatocellular carcinoma	[49]
Dysbindin		Pancreatic cancer	[50]
FBLN1	Fibulin-1	Colorectal cancer	[51]
FJX1	Four-jointed box kinase-1	Colorectal cancer	[52]
FOLR1	Folate-receptor-1	Ovarian cancer	[53]
H19	Long non-coding RNA-H19	Gastric cancer	[54]
Hsp90α	Heatshock protein 90α	Hepatocellular Carcinoma	[55,56]
ISG15	Ubiquitin-like protein ISG15	Thyroid Cancer	[57]
LG3BP	Galectin-3 recombinant protein	Lung Cancer	[45]
MIC-1	Macrophage inhibitory cytokine-1	Pancreatic Cancer	[58]
MDKN	Midkine	Neuroblastoma, Hepatocellular Carcinoma	[59,60]
miR-200	miRNA-200	Ovarian cancer	[61]
miR-200b	miRNA-200b	Hepatocellular Carcinoma	[43]
miR-21	miRNA-21	Breast Cancer	[62]
miR-421	miRNA-421,	Gastric cancer	[63]
MMP-7	Matrix metalloproteinase-7	Gastric cancer	[39]
MMP-9	Matrix metalloproteinase-9	Ovarian caner	[64]
MTA2	Metastasis-associated protein-2	Multiple myeloma	[38]
MUC5AC	Mucin 5AC	Pancreatic cancer	[65]
NY-ESO-1	New York esophageal squamous cell carcinoma,	Lung cancer	[66]
Osteopontin		Hepatocellular carcinoma	[67,68]
PA	Phosphatidic acid	Prostate cancer	[36]
Plexin-B2		Thyroid Cancer	[57]
sBCMA	Soluble B cell maturation antigen	Multiple myeloma	[69]
SOX2	SRY-Box Transcription Factor 2	Lung cancer	[66]
STK31	Serine threonine kinase-31	Colorectalcancer	[51]
TIMP-1	Tissue inhibitor metalloproteinase 1	Pancreatic cancer	[70]
TIMP4	Tissue inhibitor metalloproteinase 4	Gastric cancer	[20]
TNC	Tenascin C	Prostate Cancer	[36]
TTR	Transthyretin	Melanoma	[71]

Vitronectin	Melanoma	[48]

Table S4. Urinary Markers

Tumor Marker		Associated Cancer	Ref.
3-MTS	3-Methoxytyramine sulphate	Neuroblastoma	[72]
5-hydroxymethylcytosine		Multiple myeloma	[73]
A1-antitrypsin		Bladder cancer	[74]
APOA1	Apolipoprotein A-I	Bladder cancer	[74]
CR	Creatine riboside	Lung cancer	[75,76]
FGβ	Fibrinogen Beta Chain	Bladder cancer	[74]
NAM	nicotinic acid mononucleotide,	Thyroid Cancer	[77]
NANA	*N*-acetylneuraminic acid	Lung cancer	[75,76]
Sarcosine		Prostate cancer	[78,79]
ZA2G	Zinc-alpha-2 glycoprotein, 1	Breast Cancer	[80]

Supplementary References

1. Rossi, C.; Cicalini, I.; Cufaro, M.C.; Consalvo, A.; Upadhyaya, P.; Sala, G.; Antonucci, I.; Del Boccio, P.; Stuppia, L.; De Laurenzi, V. Breast Cancer in the Era of Integrating “Omics” Approaches. *Oncogenesis* 2022 11:1 2022, 11, 1–13, https://doi.org/0.1038/s41389-022-00393-8.
2. Alenezi, W.M.; Fierheller, C.T.; Recio, N.; Tonin, P.N. Literature Review of BARD1 as a Cancer Predisposing Gene with a Focus on Breast and Ovarian Cancers. *Genes* 2020, 11, 856, https://doi.org/10.3390/GENES11080856.
3. Tien, J.F.; Mazloomian, A.; Cheng, S.W.G.; Hughes, C.S.; Chow, C.C.T.; Canapi, L.T.; Oloumi, A.; Trigo-Gonzalez, G.; Bashashati, A.; Xu, J.; et al. CDK12 Regulates Alternative Last Exon MRNA Splicing and Promotes Breast Cancer Cell Invasion. *Nucleic Acids Res* 2017, 45, 6698–6716, https://doi.org/10.1093/NAR/GKX187.
4. Betancourt, L.H.; Gil, J.; Sanchez, A.; Doma, V.; Kuras, M.; Murillo, J.R.; Velasquez, E.; Çakır, U.; Kim, Y.; Sugihara, Y.; et al. The Human Melanoma Proteome Atlas—Complementing the Melanoma Transcriptome. *Clin Transl Med* 2021, 11, e451, https://doi.org/10.1002/CTM2.451.
5. Fargnoli, M.C.; Gandini, S.; Peris, K.; Maisonneuve, P.; Raimondi, S. MC1R Variants Increase Melanoma Risk in Families with CDKN2A Mutations: A Meta-Analysis. *Eur J Cancer* 2010, 46, 1413–1420, https://doi.org/10.1016/J.EJCA.2010.01.027.

6. Rainville, I.; Hatcher, S.; Rosenthal, E.; Larson, K.; Bernhisel, R.; Meek, S.; Gorringe, H.; Mundt, E.; Manley, S. High Risk of Breast Cancer in Women with Biallelic Pathogenic Variants in CHEK2. *Breast Cancer Res Treat* 2020, 180, 503–509, https://doi.org/10.1007/S10549-020-05543-3.
7. Chen, F.; Wang, J.; Wu, Y.; Gao, Q.; Zhang, S. Potential Biomarkers for Liver Cancer Diagnosis Based on Multi-Omics Strategy. *Front Oncol* 2022, 12, 822449, https://doi.org/10.3389/FONC.2022.822449/XML/NLM.
8. Lu, G.; Lin, J.; Song, G.; Chen, M. Prognostic Significance of CTNNB1 Mutation in Hepatocellular Carcinoma: A Systematic Review and Meta-Analysis. *Aging* 2023, 15, 9759, https://doi.org/10.18632/AGING.205047.
9. Dupont, W.D.; Breyer, J.P.; Johnson, S.H.; Plummer, W.D.; Smith, J.R. Prostate Cancer Risk Variants of the HOXB Genetic Locus. Sci Rep 2021, 11, https://doi.org/10.1038/S41598-021-89399-7.
10. Diskin, S.J.; Capasso, M.; Schnepp, R.W.; Cole, K.A.; Attiyeh, E.F.; Hou, C.; Diamond, M.; Carpenter, E.L.; Winter, C.; Lee, H.; et al. Common Variation at 6q16 within HACE1 and LIN28B Influences Susceptibility to Neuroblastoma. *Nat Genet* 2012, 44, 1126–1130, https://doi.org/10.1038/NG.2387.
11. Li, S.; Zhang, E.; Cai, Z. Liquid Biopsy by Analysis of Circulating Myeloma Cells and Cell-Free Nucleic Acids: A Novel Noninvasive Approach of Disease Evaluation in Multiple Myeloma. *Biomarker Research* 2023, 11, 1–26, https://doi.org/10.1186/S40364-023-00469-6.
12. Antoniou, A.C.; Casadei, S.; Heikkinen, T.; Barrowdale, D.; Pylkäs, K.; Roberts, J.; Lee, A.; Subramanian, D.; De Leeneer, K.; Fostira, F.; et al. Breast-Cancer Risk in Families with Mutations in PALB2 . *NEJM* 2014, 371, 497–506, https://doi.org/10.1056/NEJMOA1400382.
13. Pugh, T.J.; Morozova, O.; Attiyeh, E.F.; Asgharzadeh, S.; Wei, J.S.; Auclair, D.; Carter, S.L.; Cibulskis, K.; Hanna, M.; Kiezun, A.; et al. The Genetic Landscape of High-Risk Neuroblastoma. *Nature Genetics* 2013 45:3 2013, 45, 279–284, https://doi.org/10.1038/ng.2529.
14. Nunes-Xavier, C.E.; Zaldumbide, L.; Mosteiro, L.; López-Almaraz, R.; García de Andoin, N.; Aguirre, P.; Emaldi, M.; Torices, L.; López, J.I.; Pulido, R. Protein Tyrosine Phosphatases in Neuroblastoma: Emerging Roles as Biomarkers and Therapeutic Targets. *Front Cell Dev Biol* 2021, 9, 811297, https://doi.org/10.3389/FCELL.2021.811297.
15. Yang, X.; Song, H.; Leslie, G.; Engel, C.; Hahnen, E.; Auber, B.; Horváth, J.; Kast, K.; Niederacher, Dı.; Turnbull, C.; et al. Ovarian and Breast Cancer Risks Associated With Pathogenic Variants in RAD51C and RAD51D. *JNCI: Journal of the National Cancer Institute* 2020, 112, 1242–1250, https://doi.org/10.1093/JNCI/DJAA030.
16. Wang, Y.; Chen, P.M.; Liu, R.B., Advance in Plasma SEPT9 Gene Methylation Assay for Colorectal Cancer Early Detection. *World J Gastrointest Oncol* 2018, 10, 15–22, https://doi.org/10.4251/WJGO.V10.I1.15.
17. Gu, Y.; Ji, Y.; Jiang, H.; Qiu, G. Clinical Effect of Driver Mutations of KRAS, CDKN2A/P16, TP53, and SMAD4 in Pancreatic Cancer: A Meta-Analysis.

Genet Test Mol Biomarkers 2020, 24, 777–788, https://doi.org/10.1089/GTMB.2020.0078.
18. Hussein, R.; Abou-Shanab, A.M.; Badr, E. A Multi-Omics Approach for Biomarker Discovery in Neuroblastoma: A Network-Based Framework. *NPJ Systems Biology and Applications* 2024 10:1 2024, 10, 1–14, https://doi.org/10.1038/s41540-024-00371-3.
19. Wan, M.; Meng, H.; Li, H. Potential Role of TWIST1 and Its Methylation in Bladder Urothelial Carcinoma. *Transl Cancer Res* 2024, 13, 6070–6086, https://doi.org/10.21037/TCR-24-1029.
20. Liu, N.; Wu, Y.; Cheng, W.; Wu, Y.; Wang, L.; Zhuang, L. Identification of Novel Prognostic Biomarkers by Integrating Multi-Omics Data in Gastric Cancer. *BMC Cancer* 2021, 21, 1–16, doi:10.1186/S12885-021-08210-Y.
21. Ghazanfar, S.; Fatima, I.; Aslam, M.; Musharraf, S.G.; Sherman, N.E.; Moskaluk, C.; Fox, J.W.; Akhtar, M.W.; Sadaf, S. Identification of Actin Beta-like 2 (ACTBL2) as Novel, Upregulated Protein in Colorectal Cancer. *J Proteomics* 2017, 152, 33–40, https://doi.org/10.1016/j.jprot.2016.10.011.
22. Zalfa, F.; Perrone, M.G.; Ferorelli, S.; Laera, L.; Pierri, C.L.; Tolomeo, A.; Dimiccoli, V.; Perrone, G.; De Grassi, A.; Scilimati, A. Genome-Wide Identification and Validation of Gene Expression Biomarkers in the Diagnosis of Ovarian Serous Cystadenocarcinoma. *Cancers* 2022, 14, 3764, https://doi.org/10.3390/CANCERS14153764.
23. Han, Z.; Feng, Y.; Deng, Y.; Tang, Z.; Cai, S.; Zhuo, Y.; Liang, Y.; Ye, J.; Cai, Z.; Yang, S.; et al. Integrated Analysis Reveals Prognostic Value and Progression-Related Role of AMIGO2 in Prostate Cancer. Transl Androl Urol 2022, 11, 914–928, https://doi.org/10.21037/TAU-21-1148.
24. Jiang, J.; Ding, Y.; Wu, M.; Lyu, X.; Wang, H.; Chen, Y.; Wang, H.; Teng, L. Identification of TYROBP and C1QB as Two Novel Key Genes With Prognostic Value in Gastric Cancer by Network Analysis. *Front Oncol* 2020, 10, 533818, https://doi.org/10.3389/FONC.2020.01765.
25. Ren, H.; Liu, X.; Li, F.; He, X.; Zhao, N. Identification of a Six Gene Prognosis Signature for Papillary Thyroid Cancer Using Multi-Omics Methods and Bioinformatics Analysis. *Front Oncol* 2021, 11, 624421, https://doi.org/10.3389/FONC.2021.624421.
26. Wang, D.; Zhou, Y.; Hua, L.; Li, J.; Zhu, N.; Liu, Y. CDK3, CDK5 and CDK8 Proteins as Prognostic and Potential Biomarkers in Colorectal Cancer Patients. *Int J Gen Med* 2022, 15, 2233–2245, https://doi.org/10.2147/IJGM.S349576,.
27. Eisenstein, A.; Gonzalez, E.C.; Raghunathan, R.; Xu, X.; Wu, M.; McLean, E.O.; McGee, J.; Ryu, B.; Alani, R.M. Emerging Biomarkers in Cutaneous Melanoma. *Mol Diagn Ther* 2018, 22, 203–218, https://doi.org/10.1007/S40291-018-0318-Z,.
28. Ke, Y.; Ge, W. Identification of Prognostic Biomarkers in Neuroblastoma Using WGCNA and Multi-Omics Analysis. *Discover Oncology* 2024, 15, 1–13, https://doi.org/10.1007/S12672-024-01334-0.

29. Liu, W.; Wei, C.; He, Q.; Chen, Z.; Zhuang, W.; Guo, Y.; Xue, X. Multiple Omics Integrative Analysis Identifies GARS1 as a Novel Prognostic and Immunological Biomarker: From Pan-Cancer to Bladder Cancer. *Sci Rep* 2024, 14, 1–21, https://doi.org/10.1038/S41598-024-70041-1.
30. Piga, I.; L'Imperio, V.; Capitoli, G.; Denti, V.; Smith, A.; Magni, F.; Pagni, F. Paving the Path toward Multi-Omics Approaches in the Diagnostic Challenges Faced in Thyroid Pathology. *Expert Rev Proteomics* 2023, 20, 419–437, https://doi.org/10.1080/14789450.2023.2288222.
31. Agarwal, S.; Bychkov, A.; Jung, C.K. Emerging Biomarkers in Thyroid Practice and Research. *Cancers* 2022, 14, 204, https://doi.org/10.3390/CANCERS14010204.
32. Deng, C.; Xie, C.; Li, Z.; Mei, J.; Wang, K. Multi-Omics Analysis Identifies Diagnostic Circulating Biomarkers and Potential Therapeutic Targets, Revealing IQGAP1 as an Oncogene in Gastric Cancer. *NPJ Precision Oncology* 2025 9:1 2025, 9, 1–18, https://doi.org/10.1038/s41698-025-00895-9.
33. Chen, R.; Hu, B.; Jiang, M.; Deng, W.; Zheng, P.; Fu, B. Bioinformatic Analysis of the Expression and Clinical Significance of the DNA Replication Regulator MCM Complex in Bladder Cancer. *Int J Gen Med* 2022, 15, 5465–5485, https://doi.org/10.2147/IJGM.S368573.
34. Liao, S.; Wang, K.; Zhang, L.; Shi, G.; Wang, Z.; Chen, Z.; Zhu, P.; He, Q. PRC1 and RACGAP1 Are Diagnostic Biomarkers of Early HCC and PRC1 Drives Self-Renewal of Liver Cancer Stem Cells. *Front Cell Dev Biol* 2022, 10, 864051, https://doi.org/10.3389/FCELL.2022.864051.
35. Zygulska, A.L.; Pierzchalski, P. Novel Diagnostic Biomarkers in Colorectal Cancer. *Int J Mol Sci* 2022, 23, 852, https://doi.org/10.3390/IJMS23020852.
36. Kiebish, M.A.; Cullen, J.; Mishra, P.; Ali, A.; Milliman, E.; Rodrigues, L.O.; Chen, E.Y.; Tolstikov, V.; Zhang, L.; Panagopoulos, K.; et al. Multi-Omic Serum Biomarkers for Prognosis of Disease Progression in Prostate Cancer. *J Transl Med* 2020, 18, 1–10, https://doi.org/10.1186/S12967-019-02185-Y.
37. Lu, Y.; Li, N.; Gao, L.; Xu, Y.J.; Huang, C.; Yu, K.; Ling, Q.; Cheng, Q.; Chen, S.; Zhu, M.; et al. Acetylcarnitine Is a Candidate Diagnostic and Prognostic Biomarker of Hepatocellular Carcinoma. *Cancer Res* 2016, 76, 2912–2920, https://doi.org/10.1158/0008-5472.CAN-15-3199/652357.
38. Apipongrat, D.; Roytrakul, S.; Prayongratana, K.; Charoenpitakchai, M.; Intharanut, K.; Laoruangroj, C.; Silpsamrit, P.; Nathalang, O. Serum Proteomic Profiling Reveals MTA2 and AGO2 as Potential Prognostic Biomarkers Associated with Disease Activity and Adverse Outcomes in Multiple Myeloma. *PLoS One* 2022, 17, e0278464, https://doi.org/10.1371/JOURNAL.PONE.0278464.
39. Liu, L.; Pang, H.; He, Q.; Pan, B.; Sun, X.; Shan, J.; Wu, L.; Wu, K.; Yao, X.; Guo, Y. A Novel Strategy to Identify Candidate Diagnostic and Prognostic Biomarkers for Gastric Cancer. *Cancer Cell Int* 2021, 21, https://doi.org/10.1186/S12935-021-02007-6.

40. Lokman, N.A.; Ricciardelli, C.; Stephens, A.N.; Jobling, T.W.; Hoffmann, P.; Oehler, M.K. Diagnostic Value of Plasma Annexin A2 in Early-Stage High-Grade Serous Ovarian Cancer. *Diagnostics* 2021,11, 69, https://doi.org/10.3390/DIAGNOSTICS11010069.
41. Sun, Y.; Gao, G.; Cai, J.; Wang, Y.; Qu, X.; He, L.; Liu, F.; Zhang, Y.; Lin, K.; Ma, S.; et al. Annexin A2 Is a Discriminative Serological Candidate in Early Hepatocellular Carcinoma. *Carcinogenesis* 2013, 34, 595–604, https://doi.org/10.1093/CARCIN/BGS372.
42. Lobo, M.D.P.; Moreno, F.B.M.B.; Souza, G.H.M.F.; Verde, S.M.M.L.; Moreira, R. de A.; Monteiro-Moreira, A.C. de O. Label-Free Proteome Analysis of Plasma from Patients with Breast Cancer: Stage-Specific Protein Expression. Front Oncol 2017, 7, 218323, https://doi.org/10.3389/FONC.2017.00014.
43. Armakolas, A.; Dimopoulou, V.; Nezos, A.; Stamatakis, G.; Samiotaki, M.; Panayotou, G.; Tampaki, M.; Stathaki, M.; Dourakis, S.; Koskinas, J. Cellular, Molecular and Proteomic Characteristics of Early Hepatocellular Carcinoma. *Curr Issues Mol Biol* 2022, 44, 4714–4734, https://doi.org/10.3390/CIMB44100322.
44. Uchiyama, K.; Yagi, N.; Mizushima, K.; Higashimura, Y.; Hirai, Y.; Okayama, T.; Yoshida, N.; Katada, K.; Kamada, K.; Handa, O.; et al. Serum Metabolomics Analysis for Early Detection of Colorectal Cancer. *J Gastroenterol* 2017, 52, 677–694, https://doi.org/10.1007/S00535-016-1261-6.
45. Silvestri, G.A.; Tanner, N.T.; Kearney, P.; Vachani, A.; Massion, P.P.; Porter, A.; Springmeyer, S.C.; Fang, K.C.; Midthun, D.; Mazzone, P.J. Assessment of Plasma Proteomics Biomarker's Ability to Distinguish Benign From Malignant Lung Nodules: Results of the PANOPTIC (Pulmonary Nodule Plasma Proteomic Classifier) Trial. *Chest* 2018, 154, 491–500, https://doi.org/10.1016/J.CHEST.2018.02.012.
46. Zhu, D.; Wang, J.; Ren, L.; Li, Y.; Xu, B.; Wei, Y.; Zhong, Y.; Yu, X.; Zhai, S.; Xu, J.; et al. Serum Proteomic Profiling for the Early Diagnosis of Colorectal Cancer. *J Cell Biochem* 2013, 114, 448–455, https://doi.org/10.1002/JCB.24384.
47. Sogawa, K.; Takano, S.; Iida, F.; Satoh, M.; Tsuchida, S.; Kawashima, Y.; Yoshitomi, H.; Sanda, A.; Kodera, Y.; Takizawa, H.; et al. Identification of a Novel Serum Biomarker for Pancreatic Cancer, C4b-Binding Protein α-Chain (C4BPA) by Quantitative Proteomic Analysis Using Tandem Mass Tags. *Br J Cancer* 2016, 115, 949–956, https://doi.org/10.1038/BJC.2016.295,.
48. Ortega-Martínez, I.; Gardeazabal, J.; Erramuzpe, A.; Sanchez-Diez, A.; Cortés, J.; García-Vázquez, M.D.; Pérez-Yarza, G.; Izu, R.; Luís Díaz-Ramón, J.; de la Fuente, I.M.; et al. Vitronectin and Dermcidin Serum Levels Predict the Metastatic Progression of AJCC I–II Early-Stage Melanoma. *Int J Cancer* 2016, 139, 1598–1607, https://doi.org/10.1002/IJC.30202.
49. Shen, Q.; Fan, J.; Yang, X.R.; Tan, Y.; Zhao, W.; Xu, Y.; Wang, N.; Niu, Y.; Wu, Z.; Zhou, J.; et al. Serum DKK1 as a Protein Biomarker for the Diagnosis

of Hepatocellular Carcinoma: A Large-Scale, Multicentre Study. *Lancet Oncol* 2012, 13, 817–826, https://doi.org/10.1016/S1470-2045(12)70233-4.
50. Guo, X.; Lv, X.; Fang, C.; Lv, X.; Wang, F.; Wang, D.; Zhao, J.; Ma, Y.; Xue, Y.; Bai, Q.; et al. Dysbindin as a Novel Biomarker for Pancreatic Ductal Adenocarcinoma Identified by Proteomic Profiling. *Int J Cancer* 2016, 139, 1821–1829, https://doi.org/10.1002/IJC.30227.
51. Watany, M.M.; Elmashad, N.M.; Badawi, R.; Hawash, N. Serum FBLN1 and STK31 as Biomarkers of Colorectal Cancer and Their Ability to Noninvasively Differentiate Colorectal Cancer from Benign Polyps. *Clinica Chimica Acta* 2018, 483, 151–155, https://doi.org/10.1016/J.CCA.2018.04.038.
52. Liu, L.; Huang, Y.; Li, Y.; Wang, Q.; Hao, Y.; Liu, L.; Yao, X.; Yao, X.; Wei, Y.; Sun, X.; et al. FJX1 as a Candidate Diagnostic and Prognostic Serum Biomarker for Colorectal Cancer. *Clinical and Translational Oncology* 2022, 24, 1964–1974, https://doi.org/10.1007/S12094-022-02852-5,.
53. Leung, F.; Dimitromanolakis, A.; Kobayashi, H.; Diamandis, E.P.; Kulasingam, V. Folate-Receptor 1 (FOLR1) Protein Is Elevated in the Serum of Ovarian Cancer Patients. *Clin Biochem* 2013, 46, 1462–1468, https://doi.org/10.1016/J.CLINBIOCHEM.2013.03.010.
54. Zhou, X.; Yin, C.; Dang, Y.; Ye, F.; Zhang, G. Identification of the Long Non-Coding RNA H19 in Plasma as a Novel Biomarker for Diagnosis of Gastric Cancer. Sci Rep 2015, 5, 1–10, https://doi.org/10.1038/srep11516.
55. Liu, W.; Li, J.; Zhang, P.; Hou, Q.; Feng, S.; Liu, L.; Cui, D.; Shi, H.; Fu, Y.; Luo, Y. A Novel Pan-Cancer Biomarker Plasma Heat Shock Protein 90alpha and Its Diagnosis Determinants in Clinic. *Cancer Sci* 2019, 110, 2941–2959, https://doi.org/10.1111/CAS.14143.
56. Zhou, Y.; Deng, X.; Zang, N.; Li, H.; Li, G.; Li, C.; He, M. Transcriptomic and Proteomic Investigation of HSP90A as a Potential Biomarker for HCC. *Med Sci Monit* 2015, 21, 4039, https://doi.org/10.12659/MSM.896712.
57. Lu, H.; Pan, Y.; Ruan, Y.; Zhu, C.; Hassan, H.M.; Gao, J.; Gao, J.; Fan, L.; Liang, X.; Wang, H.; et al. Biomarker Discovery for Early Diagnosis of Papillary Thyroid Carcinoma Using High-Throughput Enhanced Quantitative Plasma Proteomics. *J Proteome Res* 2023, 22, 3200–3212, https://doi.org/10.1021/ACS.JPROTEOME.3C00187.
58. Wang, X.; Li, Y.; Tian, H.; Qi, J.; Li, M.; Fu, C.; Wu, F.; Wang, Y.; Cheng, D.; Zhao, W.; et al. Macrophage Inhibitory Cytokine 1 (MIC-1/GDF15) as a Novel Diagnostic Serum Biomarker in Pancreatic Ductal Adenocarcinoma. BMC Cancer 2014, 14, 1–11, https://doi.org/10.1186/1471-2407-14-578.
59. Ikematsu, S.; Nakagawara, A.; Nakamura, Y.; Ohira, M.; Shinjo, M.; Kishida, S.; Kadomatsu, K. Plasma Midkine Level Is a Prognostic Factor for Human Neuroblastoma. *Cancer Sci* 2008, 99, 2070–2074, https://doi.org/10.1111/J.1349-7006.2008.00957.X.
60. Gowhari, A.; Phd, S.; Fatemeh Ezzatifar, |; Aravindhan, S.; Angelina, |; Zekiy, O.; Majid, |; Phd, A.; Seyed, |; Gheibihayat, M.; et al. Shedding More Light on the Role of Midkine in Hepatocellular Carcinoma: New Perspectives on

Diagnosis and Therapy. *IUBMB Life* 2021, 73, 659–669, https://doi.org/10.1002/IUB.2458.
61. Zuberi, M.; Mir, R.; Das, J.; Ahmad, I.; Javid, J.; Yadav, P.; Masroor, M.; Ahmad, S.; Ray, P.C.; Saxena, A. Expression of Serum MiR-200a, MiR-200b, and MiR-200c as Candidate Biomarkers in Epithelial Ovarian Cancer and Their Association with Clinicopathological Features. *Clinical and Translational Oncology* 2015, 17, 779–787, https://doi.org/10.1007/S12094-015-1303-1.
62. Gao, J.; Zhang, Q.; Xu, J.; Guo, L.; Li, X. Clinical Significance of Serum MiR-21 in Breast Cancer Compared with CA153 and CEA. *Chinese Journal of Cancer Research* 2013, 25, 743, https://doi.org/10.3978/J.ISSN.1000-9604.2013.12.04.
63. Xu, Y.; Wang, G.; Hu, W.; He, S.; Li, D.; Chen, P.; Zhang, J.; Gao, Y.; Yu, D.; Zong, L. Clinical Role of MiR-421 as a Novel Biomarker in Diagnosis of Gastric Cancer Patients: A Meta-Analysis. *Medicine* 2022, 101, E29242, https://doi.org/10.1097/MD.0000000000029242.
64. Liu, C.; Shen, Y.; Tan, Q. Diagnostic and Prognostic Values of MMP-9 Expression in Ovarian Cancer: A Study Based on Bioinformatics Analysis and Meta-Analysis. *Int J Biol Markers* 2023, 38, 15–24, https://doi.org/10.1177/03936155221140421.
65. Zhang, J.; Wang, Y.; Zhao, T.; Li, Y.; Tian, L.; Zhao, J.; Zhang, J. Evaluation of Serum MUC5AC in Combination with CA19-9 for the Diagnosis of Pancreatic Cancer. *World J Surg Oncol* 2020, 18, 1–7, https://doi.org/10.1186/S12957-020-1809-Z.
66. Tang, Z.-M.; Ling, Z.-G.; Wang, C.-M.; Wu, Y.-B.; Kong, J.-L. Serum Tumor-Associated Autoantibodies as Diagnostic Biomarkers for Lung Cancer: A Systematic Review and Meta-Analysis. *PLoS One*, 2017, https://doi.org/10.1371/journal.pone.0182117.
67. Makarem, A. El Diagnostic Significance of Plasma Osteopontin in Hepatitis C Virus-Related Hepatocellular Carcinoma. *Ann Hepatol* 2011, 10, 296–305, PMID: 21677331.
68. Zhu, M.; Zheng, J.; Wu, F.; Kang, B.; Liang, J.; Heskia, F.; Zhang, X.; Shan, Y. OPN Is a Promising Serological Biomarker for Hepatocellular Carcinoma Diagnosis. *J Med Virol* 2020, 92, 3596–3603, https://doi.org/10.1002/JMV.25704.
69. Alomari, M.; Kunacheewa, C.; Manasanch, E.E. The Role of Soluble B Cell Maturation Antigen as a Biomarker in Multiple Myeloma. *Leuk Lymphoma* 2023, 64, 261–272, https://doi.org/10.1080/10428194.2022.2133540.
70. Capello, M.; Bantis, L.E.; Scelo, G.; Zhao, Y.; Li, P.; Dhillon, D.S.; Patel, N.J.; Kundnani, D.L.; Wang, H.; Abbruzzese, J.L.; et al. Sequential Validation of Blood-Based Protein Biomarker Candidates for Early-Stage Pancreatic Cancer. *J Natl Cancer Inst* 2017, 109, https://doi.org/10.1093/JNCI/DJW266,.
71. Greco, M.; Mitri, M. De; Chiriacò, F.; Leo, G.; Brienza, E.; Maffia, M. Serum Proteomic Profile of Cutaneous Malignant Melanoma and Relation to Cancer Progression: Association to Tumor Derived Alpha-N-Acetylgalactosaminidase

Activity. *Cancer Lett* 2009, 283, 222–229, https://doi.org/10.1016/J.CANLET.2009.04.001.
72. Yokota, K.; Uchida, H.; Sakairi, M.; Abe, M.; Tanaka, Y.; Tainaka, T.; Shirota, C.; Sumida, W.; Oshima, K.; Makita, S.; et al. Identification of Novel Neuroblastoma Biomarkers in Urine Samples. *Sci Rep* 2021 11:1 2021, 11, 1–9, https://doi.org/10.1038/s41598-021-83619-w.
73. Xie, W.; Li, X.; Chen, H.; Chu, J.; Zhang, L.; Tang, B.; Huang, W.; Li, L.; Lin, J.; Dong, Y. 5-Hydroxymethylcytosine Profiles of CfDNA in Urine as Diagnostic, Differential Diagnosis and Prognostic Markers for Multiple Myeloma. *Cancer Med* 2024, 13,https://doi.org/10.1002/CAM4.70477.
74. Frantzi, M.; Latosinska, A.; Flühe, L.; Hupe, M.C.; Critselis, E.; Kramer, M.W.; Merseburger, A.S.; Mischak, H.; Vlahou, A. Developing Proteomic Biomarkers for Bladder Cancer: Towards Clinical Application. *Nature Reviews Urology* 2015 12:6 2015, 12, 317–330, https://doi.org/10.1038/nrurol.2015.100.
75. Haznadar, M.; Cai, Q.; Krausz, K.W.; Bowman, E.D.; Margono, E.; Noro, R.; Thompson, M.D.; Mathé, E.A.; Munro, H.M.; Steinwandel, M.D.; et al. Urinary Metabolite Risk Biomarkers of Lung Cancer: A Prospective Cohort Study. *Cancer Epidemiol Biomarkers Prev* 2016, 25, 978–986, https://doi.org/10.1158/1055-9965.EPI-15-1191.
76. Mathé, E.A.; Patterson, A.D.; Haznadar, M.; Manna, S.K.; Krausz, K.W.; Bowman, E.D.; Shields, P.G.; Idle, J.R.; Smith, P.B.; Anami, K.; et al. Noninvasive Urinary Metabolomic Profiling Identifies Diagnostic and Prognostic Markers in Lung Cancer. *Cancer Res* 2014, 74, 3259–3270, https://doi.org/10.1158/0008-5472.CAN-14-0109.
77. Chen, J.; Hu, Q.; Hou, H.; Wang, S.; Zhang, Y.; Luo, Y.; Chen, H.; Deng, H.; Zhu, H.; Zhang, L.; et al. Metabolite Analysis-Aided Diagnosis of Papillary Thyroid Cancer. *Endocr Relat Cancer* 2019, 26, 829–841, https://doi.org/10.1530/ERC-19-0344.
78. Cernei, N.; Heger, Z.; Gumulec, J.; Zitka, O.; Masarik, M.; Babula, P.; Eckschlager, T.; Stiborova, M.; Kizek, R.; Adam, V. Sarcosine as a Potential Prostate Cancer Biomarker—A Review. International Journal of Molecular Sciences 2013, 14, 13893–13908, https://doi.org/10.3390/IJMS140713893.
79. Sreekumar, A.; Poisson, L.M.; Rajendiran, T.M.; Khan, A.P.; Cao, Q.; Yu, J.; Laxman, B.; Mehra, R.; Lonigro, R.J.; Li, Y.; et al. Metabolomic Profiles Delineate Potential Role for Sarcosine in Prostate Cancer Progression. *Nature* 2009, 457, 910–914, https://doi.org/10.1038/NATURE07762.
80. Gajbhiye, A.; Dabhi, R.; Taunk, K.; Vannuruswamy, G.; RoyChoudhury, S.; Adhav, R.; Seal, S.; Mane, A.; Bayatigeri, S.; Santra, M.K.; et al. Urinary Proteome Alterations in HER2 Enriched Breast Cancer Revealed by Multipronged Quantitative Proteomics. *Proteomics* 2016, 16, 2403–2418, https://doi.org/10.1002/PMIC.201600015.
